# Ionomycin ameliorates hypophosphatasia via rescuing alkaline phosphatase deficiency-mediated L-type Ca^2+^ channel internalization in mesenchymal stem cells

**DOI:** 10.1038/s41413-020-0090-7

**Published:** 2020-04-26

**Authors:** Bei Li, Xiaoning He, Zhiwei Dong, Kun Xuan, Wei Sun, Li Gao, Shiyu Liu, Wenjia Liu, Chenghu Hu, Yimin Zhao, Songtao Shi, Yan Jin

**Affiliations:** 1 State Key Laboratory of Military Stomatology & National Clinical Research Center for Oral Diseases & Shaanxi International Joint Research Center for Oral Diseases, Center for Tissue Engineering, School of Stomatology, The Fourth Military Medical University, Xi’an, 710032 Shaanxi China; 2 Xi’an Institute of Tissue Engineering and Regenerative Medicine, Xi’an, China; 3 Department of Pediatric Dentistry, School of Stomatology, Fourth Military Medical University, Xi’an, China; 4 Institute for Biomedical Sciences of Pain, Tangdu Hospital, Fourth Military Medical University, Xi’an, China; 5 Department of Anatomy and Cell Biology, University of Pennsylvania School of Dental Medicine, Philadelphia, PA USA; 6 South China Center of Craniofacial Stem Cell Research, Guanghua School of Stomatology, Sun Yat-sen University, 74 Zhongshan 2Rd, Guangzhou, Guangdong China

**Keywords:** Calcium and phosphate metabolic disorders, Bone

## Abstract

The loss-of-function mutations in the ALPL result in hypophosphatasia (HPP), an inborn metabolic disorder that causes skeletal mineralization defects. In adults, the main clinical features are early loss of primary or secondary teeth, osteoporosis, bone pain, chondrocalcinosis, and fractures. However, guidelines for the treatment of adults with HPP are not available. Here, we show that ALPL deficiency caused a reduction in intracellular Ca^2+^ influx, resulting in an osteoporotic phenotype due to downregulated osteogenic differentiation and upregulated adipogenic differentiation in both human and mouse bone marrow mesenchymal stem cells (BMSCs). Increasing the intracellular level of calcium in BMSCs by ionomycin treatment rescued the osteoporotic phenotype in *alpl*^+/−^ mice and BMSC-specific (*Prrx1-alpl*^−*/*−^) conditional alpl knockout mice. Mechanistically, ALPL was found to be required for the maintenance of intracellular Ca^2+^ influx, which it achieves by regulating L-type Ca^2+^ channel trafficking via binding to the α2δ subunits to regulate the internalization of the L-type Ca^2+^ channel. Decreased Ca^2+^ flux inactivates the Akt/GSK3β/β-catenin signaling pathway, which regulates lineage differentiation of BMSCs. This study identifies a previously unknown role of the ectoenzyme ALPL in the maintenance of calcium channel trafficking to regulate stem cell lineage differentiation and bone homeostasis. Accelerating Ca^2+^ flux through L-type Ca^2+^ channels by ionomycin treatment may be a promising therapeutic approach for adult patients with HPP.

## Introduction

A loss-of-function mutation in the liver/bone/kidney alkaline phosphatase (ALPL) gene results in the life-threatening disease hypophosphatasia (HPP) during early developmental periods; HPP is characterized by hypomineralization of the skeleton and teeth.^1,2^ Adult patients with HPP showed early loss of primary or secondary teeth, osteoporosis, bone pain, chondrocalcinosis, and fractures. Our previous study found age-related bone mass loss and marrow fat gain in heterozygous *Alpl*^+/−^ mice.^3^ Bone marrow mesenchymal stem cells (BMSCs) are multipotent cells capable of differentiating into various cell lineages, including osteoblasts and adipocytes. As age increases, BMSCs are more inclined to undergo differentiation into adipocytes rather than osteoblasts, resulting in an increased number of adipocytes and a decreased number of osteoblasts, which leads to osteoporosis.^4^ Our previous study also showed that ALPL governed the osteo-adipogenic balance in BMSCs and prevented cell senescence.^3^ ALPL is a ubiquitous plasma membrane-bound enzyme (ectoenzyme) that functions at physiological (neutral) and alkaline pH to hydrolyze several different molecules, including inorganic pyrophosphate,^5^ pyridoxal-5-phosphate (the active form of vitamin B6),^6^ and nucleotides.^7–9^ However, the detailed mechanism of ALPL causing age-related osteoporosis is largely unknown.

In severely affected infants with HPP, hypercalcemia, and hypercalciuria are often reported as symptoms.^10,11^ However, it is still not clear why calcium metabolism abnormalities are induced by an ALPL mutation, since ALPL plays an important role in generating inorganic phosphate. Meanwhile, whether aberrant calcium metabolism is involved in age-related osteoporosis in heterozygous *Alpl*^+/−^ mice is also unclear. It is well accepted that calcium metabolism abnormalities are closely related to calcium channels on the cell surface. Calcium influx is controlled by voltage-gated Ca^2+^ channels (VGCCs) or agonist-dependent and voltage-independent Ca^2+^ entry pathways, which are called ‘store-operated’ Ca^2+^ channels (SOCs). Changes in intracellular Ca^2+^ concentration ([Ca^2+^]_*i*_) play an essential role in regulating motility, apoptosis, differentiation, and many other cellular processes.^12^ Aberrant intracellular [Ca^2+^]_*i*_ leads to the loss of Ca^2+^ homeostasis, which causes abnormal calcium metabolism and bone disorders.^13,14^ Several types of Ca^2+^ channels are reported to regulate intracellular Ca^2+^ homeostasis in BMSCs and osteoblasts to affect bone repair.^15–17^ Thus, the regulation of Ca^2+^ channels at the membrane plays a central role in BMSC function and in bone-related diseases. However, whether ALPL modulates Ca^2+^ channels to maintain Ca^2+^ homeostasis in BMSCs is unknown.

Currently, specific medical treatment options for HPP are limited to bone-targeted enzyme replacement therapies (asfotase, Strensiq, and Alexion), which have been approved for pediatric-onset HPP.^18,19^ At this time, there are no guidelines for selecting adult patients for treatment, evaluating the results of treatment, or determining the optimal duration of treatment. In this study, we used human and mouse models to demonstrate that ALPL is required for the maintenance of intracellular Ca^2+^ influx because it regulates L-type Ca^2+^ channel trafficking via binding to the α2δ subunits, which regulate the internalization of L-type Ca^2+^ channels. This decreased Ca^2+^ flux downregulates Akt/GSK3β-mediated Wnt/β-catenin signaling in BMSCs, leading to an age-related osteoporotic phenotype. Moreover, we found that raising the intracellular level of calcium in BMSCs by treatment with ionomycin rescues the osteoporotic phenotype in *alpl*^+/−^ mice and BMSC-specific (*Prrx1-alpl*^−/−^) conditional *alpl* knockout mice, and the treatment restores stem cell function of BMSCs from HPP patients, suggesting a new strategy for HPP therapy.

## Results

### ALPL deficiency caused decreased membrane expression of L-type Ca^2+^ channels in BMSCs

**Fig. 1:**
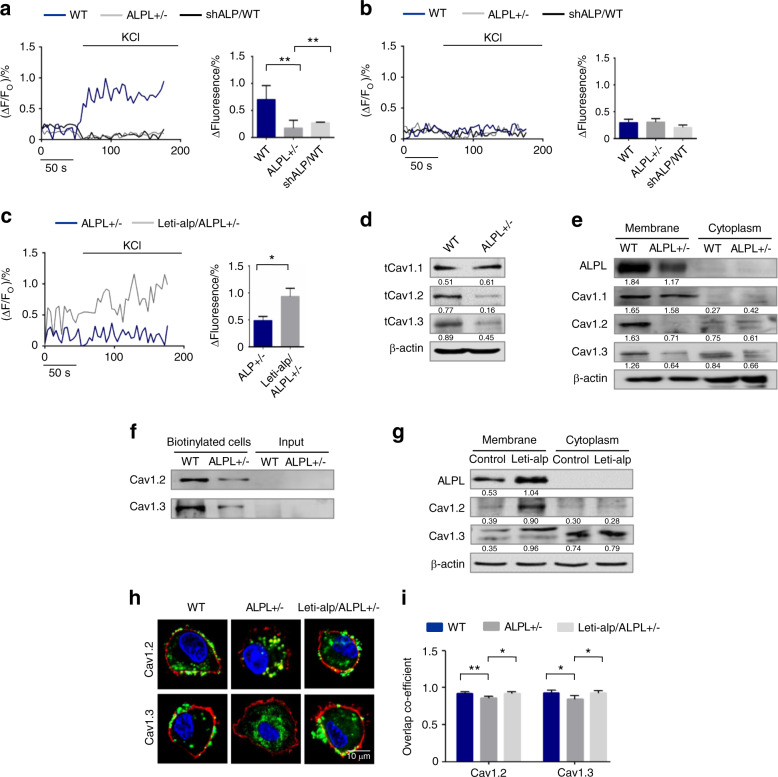
ALPL deficiency caused decreased membrane expression of L-type Ca^2+^ channels in BMSCs. **a** Ca^2+^ imaging showed decreased Ca^2+^ influx in cultured *alpl*^+/−^ BMSCs and WT BMSCs transfected with shALP (shALP/WT) after they were stimulated with 30 mmol·L^−1^ KCl for 3 min (*n* = 10). **b** No KCl-induced Ca^2+^ influx was detected in cultured WT, *alpl*^+/−^, and shALP/WT BMSCs treated with 10 mmol·L^−1^ EGTA for 3 min (*n* = 10). **c** ALPL overexpression was mediated by a lentivirus in *alpl*^+/−^ (Lenti-alp/*alpl*^+/−^) BMSCs and resulted in an elevated Ca^2+^ influx following stimulation with 30 mmol·L^−1^ KCl for 3 min (*n* = 10). **d**, **e** The expression of Ca_V_1.1, Ca_V_1.2, and Ca_V_1.3 was assessed. *alpl*^+/−^ BMSCs showed decreases in total cell expression (**d**) and membrane expression of Ca_V_1.2 and Ca_V_1.3 (**e**) and no significant change in the levels of cytoplasmic Ca_V_1.2 and Ca_V_1.3 (**e**). Total Ca_V_1.1 protein expression was not changed (**d**), and the expression of membrane and cytoplasmic Ca_V_1.1 was not altered in *alpl*^+/−^ BMSCs (**e**). **f** Cell-surface biotinylation assay. Left two lanes: western blot for Ca_V_1.2 and Ca_V_1.3 following neutravidin pull down from WT and *alpl*^+/−^ BMSCs; right two lanes: input, not biotinylated cells. **g** Lenti-alp/*alpl*^+/−^ BMSCs showed elevated membrane expression of ALP, Ca_V_1.2, and Ca_V_1.3. **h**, **i** Representative images of confocal laser scanning microscopy showing the membrane location of Ca_V_1.2 and Ca_V_1.3 (green) in WT and Lenti-alp/*alpl*^+/−^ BMSCs. The plasma membrane was stained with the marker CellMask™ Deep Red Plasma Membrane Stain (red) (**h**). Quantification of the membrane florescence was performed with NIH ImageJ (**i**). Scale bar, 10 μm. The representative results from three independent experiments are shown. Error bars represent the s.d. from the mean values. *P < 0.05; **P < 0.01; ***P < 0.001

Patients with severe ALPL deficiency develop hypercalcemia,^10,11^ so we examined the plasma calcium level in *alpl*^+/−^ mice and found a marked increase in the level of plasma calcium (Fig. S1a). To explore whether ALPL deficiency contributes to abnormal calcium metabolism in BMSCs, we isolated BMSCs from *alpl*^+/−^ mice (Fig. S1b–e) and examined cytosolic Ca^2+^. We found that introducing a lentivirus that overexpressed ALPL (Fig. S2a) into *alpl*^+/−^ BMSCs was able to increase the cytosolic Ca^2+^ of *alpl*^+/−^ BMSCs (Fig. S2b). Ca^2+^ entry across the plasma membrane occurs via two distinct kinds of channels, SOCs, and VGCCs. To test which type of Ca^2+^ channels might be affected in the absence of ALPL, WT, and *alpl*^+/−^ BMSCs were cultured with 10 nmol·L^−1^ thapsigargin (TG). TG is a noncompetitive inhibitor capable of raising the cytosolic Ca^2+^ concentration via blocking the ability of the cell to pump Ca^2+^ into the sarcoplasmic and endoplasmic reticula to activate plasma membrane Ca^2+^ channels. No significant differences were evident in TG-induced intracellular Ca^2+^ influx that were detected in WT, *alpl*^+/−^, and shALP-treated BMSCs (Fig. S2c, d). These data suggest that ALPL deficiency affects VGCC function in BMSCs. The contribution of ALPL to the function of VGCCs in BMSCs was determined after membrane depolarization using 30 mmol·L^−1^ KCl. Intracellular Ca^2+^ imaging analysis showed that KCl-induced Ca^2+^ influx was significantly decreased in culture-expanded *alpl*^+/−^ and shALP-treated BMSCs compared with that of WT BMSCs (Fig. 1a). Moreover, intracellular Ca^2+^ imaging analysis showed that KCl-induced Ca^2+^ influx was not changed in culture-expanded WT, *alpl*^+/−^, and shALP-treated BMSCs when treated with 10 mmol·L^−1^ EGTA (Fig. 1b), suggesting that ALPL causes Ca^2+^ elevation mainly due to Ca^2+^ influx with a limited contribution from intracellular Ca^2+^ storage. Overexpression of ALPL in *alpl*^+/−^ BMSCs rescued KCl-induced Ca^2+^ influx (Fig. 1c).

The decrease in Ca^2+^ currents in *alpl*^+/−^ BMSCs could arise from the loss of channels at the membrane. The VGCCs comprise ten subsets: Ca_V_1.1, Ca_V_1.2, Ca_V_1.3, Ca_V_1.4 (L-type), Ca_V_2.1 (P/Q-type), Ca_V_2.2 (N-type), Ca_V_2.3 (R-type), Ca_V_3.1, Ca_V_3.2, and Ca_V_3.3 (T-type), which are encoded by cacna1s, cacna1c, cacna1d, cacna1f, cacna1a, cacna1b, cacna1e, cacna1g, cacna1h, and cacna1i, respectively.^20,21^ To identify which subunits of the VGCCs were regulated by ALPL, we measured the total protein expression of Ca_V_1.1, Ca_V_1.2, Ca_V_1.3, Ca_V_2.1, Ca_V_2.2, Ca_V_2.3, Ca_V_3.1, Ca_V_3.2, and Ca_V_3.3, as Ca_V_1.4 expression seems to be restricted to the retina. The results showed that the total protein expression of Ca_V_1.2 and Ca_V_1.3 was decreased significantly in *alpl*^+/−^ BMSCs compared with WT BMSCs (Fig. 1d). Total protein expression of Ca_V_1.1, Ca_V_2.2, Ca_V_2.3, and Ca_V_3.3 was not changed (Figs. 1d and S2e). However, total and membrane expression levels of Ca_V_2.1, Ca_V_3.1, and Ca_V_3.2 were increased in *alpl*^+/−^ BMSCs compared with WT BMSCs (Fig. S2e, f). The expression of Ca_V_2.1, Ca_V_3.1, and Ca_V_3.2 in the cytoplasm was not changed significantly in *alpl*^+/−^ BMSCs compared with that of WT BMSCs (Fig. S2g). We used a plasma membrane protein extraction kit (Abcam, ab65400) to isolate plasma membrane protein, and then we measured the expression levels of membrane Ca_V_1.1, Ca_V_1.2, and Ca_V_1.3. The results showed that *alpl*^+/−^ BMSCs expressed similar levels of Ca_V_1.1 in the membrane and cytoplasm to what was observed in WT BMSCs (Fig. 1e). Considering the decreased Ca^2+^ influx in *alpl*^+/−^ BMSCs, we focused on Ca_V_1.2 and Ca_V_1.3. Membrane expression of calcium channels affects calcium influx, and we compared the expression of Ca_V_1.2 and Ca_V_1.3 in the membrane and in the cytoplasm. The results showed that the expression levels of membrane, Ca_V_1.2 and Ca_V_1.3 were decreased in *alpl*^+/−^ BMSCs, but the cytoplasmic levels did not change (Fig. 1e). We also measured KCl-induced Ca^2+^ influx after knockdown of Ca_V_1.2 or Ca_V_1.3 in WT BMSCs. The results showed that knockdown of Ca_V_1.2 or Ca_V_1.3 reduced KCl-induced Ca^2+^ influx in WT BMSCs (Fig. S2h). Moreover, we labeled the surface molecules with biotin and used neutravidin ultralink resin beads to capture biotinylated surface proteins. Then, we used western blot analysis to show the expression levels of proteins captured by anti-Ca_V_1.2 and Ca_V_1.3 antibodies. The results showed that the amount of Ca_V_1.2 or Ca_V_1.3 on the cell surface was decreased in *alpl*^+/−^ BMSCs compared with that of the WT BMSCs (Fig. 1f). However, overexpression of ALPL increased the expression of Ca_V_1.2 and Ca_V_1.3 on the membrane, as assayed by western blot (Fig. 1g). We also investigated the membrane localization of Ca_V_1.2 and Ca_V_1.3 in WT and *alpl*^+/−^ BMSCs by confocal laser scanning microscopy. The results showed that Ca_V_1.2 (FITC-labeled) and Ca_V_1.3 (FITC-labeled) were localized to the membrane (as visualized by CellMask™ Deep Red Plasma Membrane Stain) of WT BMSCs (Fig. 1h, i). However, Ca_V_1.2 and Ca_V_1.3 were absent from the membrane of *alpl*^+/−^ BMSCs. Ca_V_1.2 and Ca_V_1.3 were localized to the cell membrane after overexpression of ALPL in *alpl*^+/−^ BMSCs (Fig. 1h, i). These results suggest that ALPL modulates the expression of L-type Ca^2+^ channels, especially Ca_V_1.2 and Ca_V_1.3.

### ALPL-regulated MSC osteogenic/adipogenic lineage differentiation via the L-type Ca^2+^ channel

**Fig. 2:**
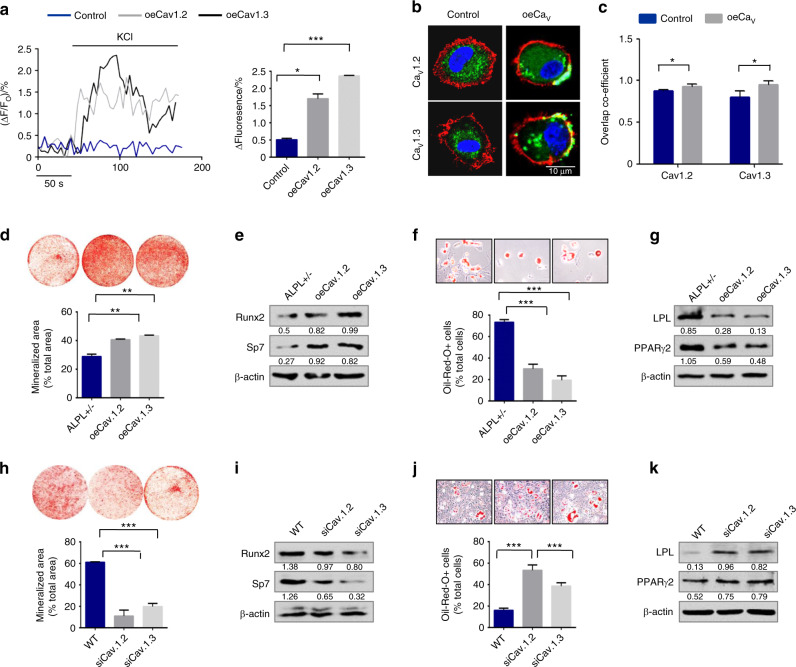
ALPL-maintained MSC osteogenic/adipogenic lineage differentiation ability via the L-type Ca^2+^ channel. **a** Ca^2+^ imaging showed elevated Ca^2+^ influx in *alpl*^+/−^ BMSCs transfected with oeCa_V_1.2 or oeCa_V_1.3 following stimulation with 30 mmol·L^−1^ KCl for 3 min (*n* = 10). **b**, **c** Representative images of confocal laser scanning microscopy showing the membrane location of Ca_V_1.2 and Ca_V_1.3 (green) in *alpl*^+/−^ BMSCs transfected with oeCa_V_1.2 or oeCa_V_1.3. The plasma membrane was stained with the marker CellMask™ Deep Red Plasma Membrane Stain (red) (**b**). Quantification of the membrane florescence was performed with NIH ImageJ (**c**). Scale bar, 10 μm. Alizarin red staining showed that *alpl*^+/−^ BMSCs transfected with oeCa_V_1.2 or oeCa_V_1.3 had an increased capacity to form mineralized nodules when cultured under osteoinductive conditions (**d**) and they exhibited an upregulation of the osteogenic-related proteins RUNX2 and Sp7 (**e**). oeCa_V_1.2- or oeCa_V_1.3-treated *alpl*^+/−^ BMSCs showed a decreased number of oil red O-positive adipocytes when cultured under adipo-inductive conditions (**f**) and there was a downregulation of the adipogenic-related proteins PPARγ2 and LPL, as assessed by western blot (**g**). **h** Alizarin red staining showed that *alpl*^+/−^ BMSCs transfected with siCa_V_1.2 or siCa_V_1.3 had a decreased capacity to form mineralized nodules when cultured under osteoinductive conditions. **i** Western blot analysis showed that BMSCs transfected with siCa_V_1.2 or siCa_V_1.3 expressed decreased levels of the osteogenic-related proteins RUNX2 and Sp7. β-actin was used as a protein loading control. BMSCs transfected with siCa_V_1.2 or siCa_V_1.3 showed an increased number of oil red O-positive adipocytes when cultured under adipo-inductive conditions (**j**) and upregulation of the adipogenic-related proteins PPARγ2 and LPL, as assessed by western blot (**k**). The representative results from three independent experiments are shown. Error bars represent the s.d. from the mean values. *P < 0.05; **P < 0.01; ***P < 0.001

We knocked down ALPL in BMSCs by siRNA treatment (Fig. S3a) and confirmed that ALPL deficiency decreased the osteogenic differentiation and increased the adipogenic differentiation of BMSCs (Fig. S3b–e). To explore the function of long-lasting voltage-gated calcium channel (L-VGCC) in the osteogenic/adipogenic differentiation of BMSCs, we used L-type Ca^2+^ channel blockers, diltiazem, or nifedipine, to treat WT BMSCs, and we explored their resulting osteogenic and adipogenic differentiation capacities (Fig. S3f–i). We found that both nifedipine and diltiazem inhibited the osteogenic differentiation ability of BMSCs, as evidenced by decreases in mineralized nodule formation and in expression of the osteogenic markers Bglap, Ibsp, RUNX2, and Sp7. In addition, nifedipine promoted the adipogenic differentiation of BMSCs, whereas diltiazem inhibited the adipogenic differentiation of BMSCs, as assessed by oil red O staining. The western blot assay data showed the same changes in the adipogenic regulators PPARγ2 and LPL under adipogenic culture conditions. To further address whether the defect in osteogenic/adipogenic lineage differentiation in ALPL-deficient BMSCs was due to the abnormal membrane expression of VGCCs, especially Ca_V_1.2 and Ca_V_1.3, we overexpressed Ca_V_1.2 and Ca_V_1.3 in *alpl*^+/−^ BMSCs (oeCa_V_1.2 or oeCa_V_1.3) (Fig. S3j). Western blot data showed that Ca_V_1.2 and Ca_V_1.3 were increased on the membrane of *alpl*^+/−^ BMSCs after Ca_V_1.2 or Ca_V_1.3 overexpression (oeCa_V_1.2 or oeCa_V_1.3) (Fig. S3k). KCl-induced Ca^2+^ influx was elevated after overexpression of Ca_V_1.2 or Ca_V_1.3 in *alpl*^+/−^ BMSCs (Fig. 2a). We also observed the membrane localization of Ca_V_1.2 and Ca_V_1.3 in *alpl*^+/−^ BMSCs after overexpressing Ca_V_1.2 or Ca_V_1.3 by confocal laser scanning microscopy (Fig. 2b, c). We found that overexpression of Ca_V_1.2 or Ca_V_1.3 in *alpl*^+/−^ BMSCs (oeCa_V_1.2 or oeCa_V_1.3) rescued the decreased osteogenic differentiation of BMSCs, as evidenced by increases in mineralized nodule formation and in expression of the osteogenic markers RUNX2 and Sp7 (Fig. 2d, e). In contrast, overexpression of Ca_V_1.2 or Ca_V_1.3 (oeCa_V_1.2 or oeCa_V_1.3) decreased adipogenic differentiation of BMSCs, as assessed by oil red O staining, showing decreased numbers of adipocytes; further, western blots showed downregulation of the adipogenic regulators PPARγ2 and LPL under adipogenic culture conditions (Fig. 2f, g). However, knockdown of Ca_V_1.2 or Ca_V_1.3 (by treatment with siCa_V_1.2 or siCa_V_1.3, respectively) (Fig. S3l, m) in WT BMSCs caused decreased osteogenic and increased adipogenic differentiation (Fig. 2h–k). These data indicate that ALPL regulates osteogenic and adipogenic lineage differentiation through Ca_V_1.2- and Ca_V_1.3-mediated calcium influx.

### ALPL deficiency promoted the internalization of L-type Ca^2+^ channels in BMSCs

**Fig. 3:**
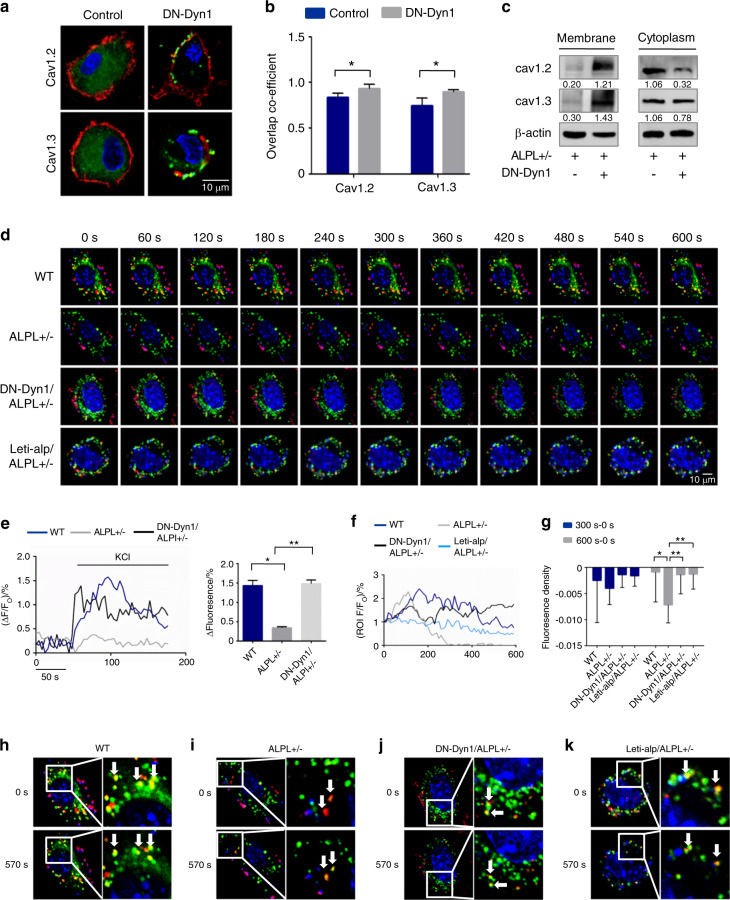
ALPL deficiency promoted the internalization of L-type Ca^2+^ channels in BMSCs. **a**, **b** Representative images of confocal laser scanning microscopy showing the membrane location of Ca_V_1.2 and Ca_V_1.3 (green) in *alpl*^+/−^ BMSCs transfected with DN-Dyn1. The plasma membrane was stained with the marker CellMask™ Deep Red Plasma Membrane Stain (red) (**a**). Quantification of the membrane florescence was performed with NIH ImageJ (**b**). Scale bar, 10 μm. **c** *alpl*^+/−^ BMSCs transfected with DN-Dyn1 showed upregulation of Ca_V_1.2 and Ca_V_1.3 membrane expression and almost no change in the cytoplasmic expression of Ca_V_1.2 and Ca_V_1.3, as assessed by western blot. β-actin was used as a protein loading control. **d** 10-min time-lapse confocal laser scanning microscopy images of WT, *alpl*^+/−^, DN-Dyn1/*alpl*^+/−^, and Lenti-alp/*alpl*^+/−^ BMSCs containing DsRed-CaV1.2. Scale bar, 10 μm. **e** Ca^2+^ imaging showed elevated Ca^2+^ influx of cultured *alpl*^+/−^ BMSCs transfected with DN-Dyn1 after stimulation with 30 mmol·L^−1^ KCl for 3 min (*n* = 10). *alpl*^+/−^ BMSCs showed a pronounced decrease in DsRed-Ca_V_1.2 at the membrane (Dio-labeled ROI, *n* = 10) (**f**). **g** Quantification of the florescence in the ROI during the time-course lapse at 0 s, 300 s, and 600 s. **h**–**k** Representative images show the colocalization of DsRed-Ca_V_1.2 with the Dio-labeled cell membrane of BMSCs. WT, DN-Dyn1/*alpl*^+/−^, and Lenti-alp/*alpl*^+/−^ BMSCs had colocalized regions at 0 s and 570 s (**h**, **j**, **k**). However, *alpl*^+/−^ BMSCs showed no colocalization at 0 s and 570 s (**i**). The representative results from three independent experiments are shown. Error bars represent the s.d. from the mean values. *P < 0.05; **P < 0.01

To determine whether a lack of ALPL leads to channel internalization resulting in decreased membrane expression of L-type Ca^2+^ channels in BMSCs, we disrupted endocytosis by expressing a dominant-negative mutant of dynamin 1 (DN-Dyn1), which is a GTPase required for the formation of endocytic vesicles from the plasma membrane.^22^ The expression of DN-Dyn1 in *alpl*^+/−^ BMSCs prevented the loss of Ca_V_1.2 and Ca_V_1.3 on the cell surface (Fig. 3a, b), providing evidence that a lack of ALPL causes internalization of the channels. Western blot analysis showed that the expression levels of membrane Ca_V_1.2 and Ca_V_1.3 in *alpl*^+/−^ BMSCs were increased after DN-Dyn1 transfection. However, the expression levels of cytoplasmic Ca_V_1.2 and Ca_V_1.3 in *alpl*^+/−^ BMSCs were decreased after DN-Dyn1 transfection (Fig. 3c). The expression of DN-Dyn1 also prevented the decrease in KCl-induced Ca^2+^ influx in *alpl*^+/−^ BMSCs (Fig. 3e).

We next measured the time course of ALPL-dependent L-type Ca^2+^ channel internalization. To study this process in live BMSCs, we used Dio to label the cell membrane (FITC-labeled) and constructed a plasmid to express Ca_V_1.2 (DsRed-Cav1.2) to use in the transfection of cells. We recorded colocalization regions as region of interest (ROI) to record the time-course change of intensity of red fluorescence, and we quantified the red florescence in each ROI over a time-course lapse at 300 s minus 0 s and 600 s minus 0 s to determine changes during the process. The DsRed-Ca_V_1.2 signal in *alpl*^+/−^ BMSCs declined significantly after 10 min compared with that of the WT, DN-Dyn1-transfected *alpl*^+/−^ BMSCs, and ALPL-overexpressed *alpl*^+/−^ BMSCs, reflecting the decreased membrane expression of the L-type Ca^2+^ channel (Fig. 3d, f and g). We also selected images at 0 s and 570 s to show the colocalization of DsRed-Ca_V_1.2 with the cell membrane of BMSCs. Almost no region of colocalization was found in *alpl*^+/−^ BMSCs (Fig. 3h–k), which suggested that ALPL deficiency promoted the internalization of L-type Ca^2+^ channels. To determine whether the expression of DN-Dyn1 rescues differentiation in ALPL-deficient BMSCs, we performed osteogenic and adipogenic induction after transfection. Expression of DN-Dyn1 increased osteogenic differentiation of *alpl*^+/−^ BMSCs, as evidenced by increased mineralized nodule formation and expression of the osteogenic markers RUNX2 and Sp7 (Fig. S4a, b). In contrast, expression of DN-Dyn1 decreased adipogenic differentiation of *alpl*^+/−^ BMSCs, as assessed by oil red O staining, which showed decreased numbers of adipocytes, and western blotting, which showed downregulation of the adipogenic regulators PPARγ2 and LPL under adipogenic culture conditions (Fig. S4c, d).

### ALPL deficiency promoted the internalization of L-type Ca^2+^ channels via binding to α2δ subunits

**Fig. 4:**
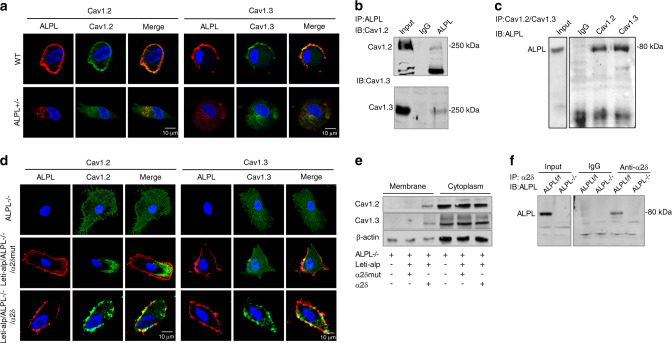
ALPL deficiency promoted the internalization of L-type Ca^2+^ channels via binding to α2δ subunits. **a** Representative images of confocal laser scanning microscopy showing a region of membrane colocalization for ALPL (DsRed) and Ca_V_1.2 (FITC-labeled) or Ca_V_1.3 (FITC-labeled) in WT BMSCs. No region of membrane colocalization was found in *alpl*^+/−^ BMSCs. Scale bar, 10 μm. **b** ALPL immunoprecipitated Ca_V_1.2 and Ca_V_1.3. The left lane shows the expression of Ca_V_1.2 and Ca_V_1.3, and the right lane shows the levels of Ca_V_1.2 and Ca_V_1.3 following immunoprecipitation with an anti-ALPL antibody. **c** Ca_V_1.2 and Ca_V_1.3 immunoprecipitated ALPL. The left panel shows the expression of ALPL, and the right panel shows the level of ALPL following immunoprecipitation with anti-Ca_V_1.2 or anti-Ca_V_1.3 antibodies. **d** Representative images of confocal laser scanning microscopy showing the membrane colocalization region of ALPL (Cy3-labeled) and Ca_V_1.2 (FITC-labeled) or Ca_V_1.3 (FITC-labeled) in *alpl*^−/−^ BMSCs overexpressing ALPL and the α2δ subunit. No membrane colocalization region was found in *alpl*^−/−^ BMSCs and *alpl*^−/−^ BMSCs overexpressing ALPL or the mutant α2δ subunit. Scale bar, 10 μm. **e** Western blot analysis showed membrane expression of Ca_V_1.2 or Ca_V_1.3 in *alpl*^−/−^ BMSCs overexpressing ALPL and the α2δ subunit. No membrane expression of Ca_V_1.2 or Ca_V_1.3 was found in *alpl*^−/−^ BMSCs and *alpl*^−/−^ BMSCs overexpressing ALPL or the mutant α2δ subunit. No significant change in cytoplasmic Ca_V_1.2 or Ca_V_1.3 was found in *alpl*^−/−^ BMSCs, *alpl*^−/−^ BMSCs overexpressing ALPL and the mutant α2δ subunit, or *alpl*^−/−^ BMSCs overexpressing ALPL and the α2δ subunit. **f** α2δ immunoprecipitated ALPL. The left panel shows the expression of ALPL, and the right panel shows the level of ALPL following immunoprecipitation with an anti-α2δ antibody in *alp/*^*f/f*^ and *alpl*^−/−^ BMSCs. β-actin was used as a protein loading control. The representative results from three independent experiments are shown

Given that ALPL has been reported to hydrolyze inorganic pyrophosphate (PPi) and adenosine triphosphate (ATP), we compared the expression of Ca_V_1.2 and Ca_V_1.3 in BMSCs and BMSCs treated with PPi or ATP. However, the addition of exogenous PPi or ATP hardly changed the membrane expression of Ca_V_1.2 and Ca_V_1.3 in BMSCs (Fig. S5a). To further explore the molecular mechanism of ALPL-regulated internalization of Ca_V_1.2 and Ca_V_1.3, we measured the membrane localization of ALPL and calcium channels. We found that Ca_V_1.2 and Ca_V_1.3 overlapped with ALPL in BMSCs (Fig. 4a, upper panel), suggesting the association of ALPL and L-type Ca^2+^ channels. However, no membrane location of Ca_V_1.2 and Ca_V_1.3 was found in *alpl*^+/−^ BMSCs (Fig. 4a, lower panel). Several regions of ALPL and Ca_V_1.2 and Ca_V_1.3 colocalization were found in the cytoplasm of *alpl*^+/−^ BMSCs (Fig. 4a, lower panel), indicating that ALPL may bind L-type Ca^2+^ channels in the cytoplasm. We also used immunoprecipitation to confirm the association of ALPL and the L-type Ca^2+^ channel. Immunoprecipitation using a control antibody did not isolate either protein, but immunoprecipitation with anti-ALPL resulted in coimmunoprecipitation with Ca_V_1.2 or Ca_V_1.3 (Fig. 4b). In addition, we found that anti-Ca_V_1.2 or anti-Ca_V_1.3 immunoprecipitated ALPL (Fig. 4c), suggesting that ALPL associates with Ca_V_1.2 and Ca_V_1.3 in BMSCs.

We next investigated which regions of Ca_V_1.2 and Ca_V_1.3 are important for ALPL-regulated internalization. The α_2_δ subunit has been reported to traffic α1 subunits, which influences internalization of the channels.^23,24^ Floxed *alpl* mice with *Prrx1*::*Cre* mice were crossed to generate early embryonic MSC-specific (*Prrx1-alpl*^−/−^) conditional *alpl* knockout mice (Fig. S5b, c). We isolated BMSCs from the *Prrx1-alpl*^−/−^ mice (ALPL^*−/−*^) and control *alpl*^*f/f*^ littermates (control)*. Alpl*^−/−^ BMSCs showed decreased expression of ALPL, and treatment with a lentivirus that overexpressed ALPL (Lenti-alp) elevated the membrane expression of ALPL (Fig. S5d). To explore whether ALPL interacted with α_2_δ to regulate the internalization of Ca_V_1.2 and Ca_V_1.3, we expressed ALPL with α_2_δ or a mutant α_2_δ in *alpl*^−/−^ BMSCs and examined the membrane expression of Ca_V_1.2 and Ca_V_1.3. *alpl*^−/−^ BMSCs were isolated from *Prrx1-alpl*^−/−^ mice and showed no expression of ALPL, Ca_V_1.2, or Ca_V_1.3 at the membrane (Fig. 4d). The membrane expression of Ca_V_1.2 and Ca_V_1.3 was increased, and ALPL was colocalized with Ca_V_1.2 and Ca_V_1.3 after transfection with ALPL and α_2_δ (Fig. 4d). However, the membrane expression of Ca_V_1.2 and Ca_V_1.3 was not increased, and ALPL was not colocalized with Ca_V_1.2 and Ca_V_1.3 after transfection with ALPL and mutant α_2_δ (Fig. 4d). Western blot analysis also confirmed that the membrane expression of Ca_V_1.2 and Ca_V_1.3 was increased after transfection with ALPL and α_2_δ (Fig. 4e). However, no membrane expression of Ca_V_1.2 and Ca_V_1.3 was found in *alpl*^−/−^ BMSCs and *alpl*^−/−^ BMSCs transfected with ALPL and mutant α_2_δ (Fig. 4e). Immunoprecipitation using a control antibody did not isolate either protein in WT or *alpl*^−/−^ BMSCs, but immunoprecipitation with anti-α2δ in *alpl*^*f/f*^ BMSCs isolated ALPL (Fig. 4f). To confirm that ALPL interacts with α_2_δ subunits and thus regulates the lineage differentiation of BMSCs, we transfected *alpl*^+/−^ BMSCs with α_2_δ or mutant α_2_δ and assessed their osteogenic or adipogenic induction. *alpl*^+/−^ BMSCs transfected with mutant α_2_δ showed decreased osteogenic differentiation and increased adipogenic differentiation compared with *alpl*^+/−^ BMSCs transfected with α_2_δ (Fig. S5e–h).

### ALPL deficiency caused aberrant lineage differentiation of BMSCs through the Wnt/β-catenin pathway

**Fig. 5:**
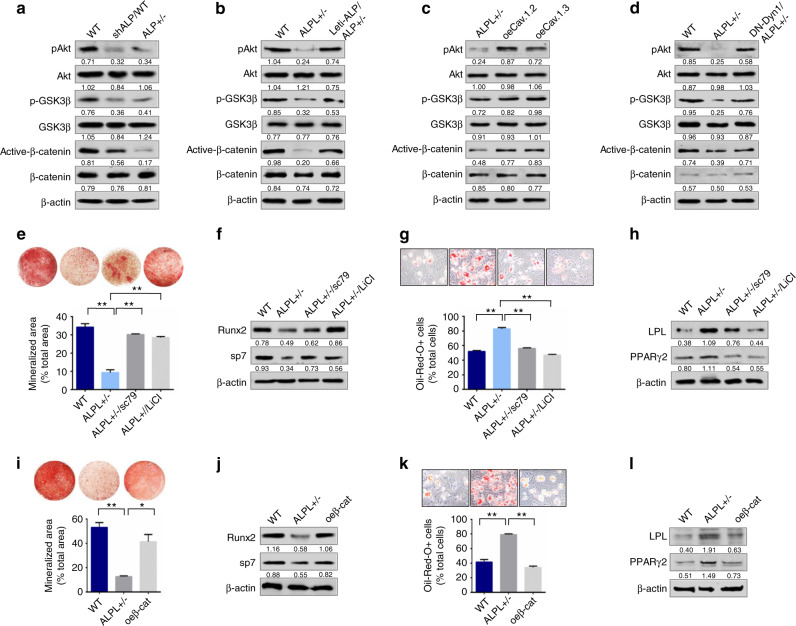
ALPL deficiency caused an imbalance in lineage differentiation of BMSCs through the Wnt/β-catenin pathway. **a** *alpl*^+/−^ and shALP BMSCs showed significant downregulation of p-AKT, p-GSK3β, and active β-catenin compared with what was observed in WT BMSCs. β-actin was used as a protein loading control. **b** Lenti-alp/*alpl*^+/−^ BMSCs showed significant upregulation of p-AKT, p-GSK3β, and active β-catenin compared with that of *alpl*^+/−^ BMSCs. **c** *alpl*^+/−^ BMSCs transfected with oeCa_V_1.2 or oeCa_V_1.3 showed significant upregulation of p-AKT, p-GSK3β, and active β-catenin. **d** *alpl*^+/−^ BMSCs transfected with DN-Dyn1 showed significant upregulation of p-AKT, p-GSK3β, and active β-catenin. Alizarin red staining revealed that *alpl*^+/−^ BMSCs treated with 10 μmol·L^−1^ sc79 or 10 mmol·L^−1^ LiCl had an increased capacity to form mineralized nodules when cultured under osteoinductive conditions (**e**), and they exhibited upregulation of the osteogenic-related proteins RUNX2 and Sp7 (**f**). *alpl*^+/−^ BMSCs treated with sc79 or LiCl showed a decreased number of oil red O-positive adipocytes when cultured under adipo-inductive conditions (**g**), and they exhibited downregulation of the adipogenic-related proteins PPARγ2 and LPL, as assessed by western blot (**h**). Alizarin red staining showed that *alpl*^+/−^ BMSCs transfected with a plasmid overexpressing β-catenin (oeβ-cat/ALPL^+/−^) had an increased capacity to form mineralized nodules when cultured under osteoinductive conditions (**i**), and they exhibited an upregulation in the osteogenic-related proteins RUNX2 and Sp7 (**j**). *alpl*^+/−^ BMSCs transfected with a plasmid overexpressing β-catenin (oeβ-cat/ALPL^+/−^) showed a decreased number of oil red O-positive adipocytes when cultured under adipo-inductive conditions (**k**), and they exhibited downregulation of the adipogenic-related proteins PPARγ2 and LPL, as assessed by western blot (**l**). The representative results from three independent experiments are shown. Error bars represent the s.d. from the mean values. *P < 0.05; **P < 0.01

To examine how ALPL deficiency-induced reduction of Ca^2+^ influx affects the osteogenic and adipogenic differentiation of BMSCs, we analyzed three Ca^2+^ downstream pathways (PKC/Erk, PI3K/Akt/GSK3β, and CaMKII/calcineurine A), which are closely linked to Ca^2+^-associated regulation of osteogenic differentiation. We found that the expression level of p-Akt significantly decreased along with the reduction of p-GSK3β in *alpl*^+/−^ BMSCs and BMSCs transfected with shALP (Fig. 5a). However, the PKC/Erk and CaMKII/calcineurine A pathways were not changed significantly in *alpl*^+/−^ BMSCs or in BMSCs transfected with shALP compared with that of WT BMSCs (Fig. S6a). Because the decrease in GSK3β phosphorylation inhibits the nuclear translocation of β-catenin, which regulates the osteogenic and adipogenic differentiation of BMSCs, we examined the expression levels of total and active β-catenin. We found that the expression of active β-catenin was decreased in both *alpl*^+/−^ BMSCs and BMSCs transfected with shALP (Fig. 5a). Moreover, when we overexpressed ALPL in *alpl*^+/−^ BMSCs, the expression of p-Akt, p-GSK3β, and active β-catenin was increased to levels similar to those of the WT BMSCs (Fig. 5b). When we overexpressed Ca_V_1.2 or Ca_V_1.3 in *alpl*^+/−^ BMSCs, the expression of p-Akt, p-GSK3β, and active β-catenin was increased (Fig. 5c). In addition, the expression of DN-Dyn1 in *alpl*^+/−^ BMSCs also increased the expression of p-Akt, p-GSK3β, and active β-catenin compared with *alpl*^+/−^ BMSCs (Fig. 5d). Taken together, these data indicate that ALPL regulates Ca^2+^ influx to affect p-Akt and p-GSK3β expression and subsequently targets the Wnt/β-catenin pathway in BMSCs.

To further determine whether ALPL regulates osteogenic and adipogenic differentiation through the Akt/GSK3β/Wnt/β-catenin pathway, we used activators of Akt phosphorylation (sc79) and GSK3β phosphorylation (LiCl) (Fig. S6b) to treat *alpl*^+/−^ BMSCs, and we found that sc79 and LiCl treatment increased osteogenic differentiation, as evidenced by increases in mineralized nodule formation and in expression of RUNX2 and Sp7 (Fig. 5e, f). In contrast, sc79 and LiCl treatment decreased adipogenic differentiation of *alpl*^+/−^ BMSCs, as assessed by oil red O staining, which showed a decreased number of adipocytes, and western blotting, which indicated a downregulation of PPARγ2 and LPL under adipogenic culture conditions (Fig. 5g, h). Moreover, we also overexpressed β-catenin (oeβ-cat) in *alpl*^+/−^ BMSCs (Fig. S6c) and observed a recovery of lineage differentiation in *alpl*^+/−^ BMSCs, as evidenced by the increased osteogenic differentiation and decreased adipogenic differentiation (Fig. 5i–l).

### Raising the intracellular level of calcium by ionomycin rescued ALPL deficiency-induced age-related osteoporosis

**Fig. 6:**
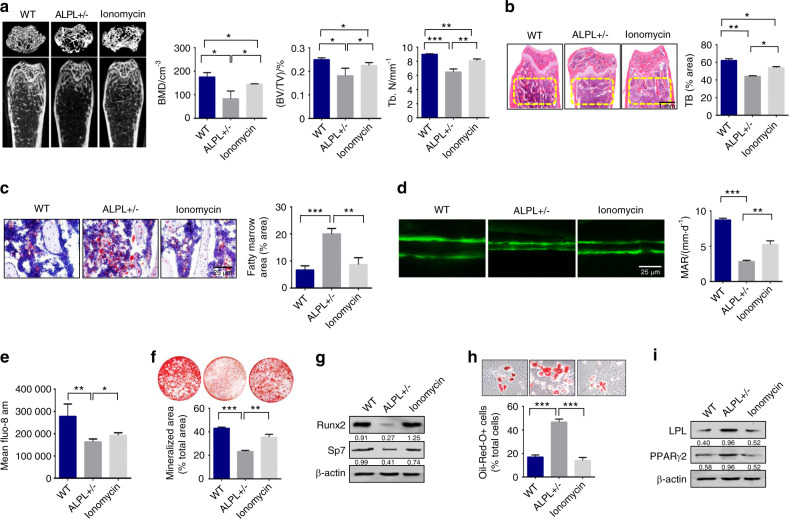
Raising the intracellular level of calcium by ionomycin rescued ALPL deficiency-induced age-related osteoporosis. **a** MicroCT analysis showed that *alpl*^+/−^ mice (*n* = 12) had significantly decreased bone mineral density (BMD), Bone volume relative to tissue volume (BV/TV), and trabecular number (Tb.N) in trabecular bone (TB) of the distal femur compared the levels in WT littermates and *alpl*^+/−^ mice treated with ionomycin at a dose of 1 mg/kg/day for 28 days (*n* = 10). **b** H&E staining showed a decreased TB volume (yellow circled area) in the distal femurs of *alpl*^+/−^ mice compared with the WT group and *alpl*^+/−^ mice treated with ionomycin. Scale bar, 1 mm. **c** Representative histological images of distal femurs showed a significantly increased number of adipocytes in *alpl*^+/−^ mouse bone marrow, as assessed by oil red O staining. Scale bar, 25 μm. **d** The calcein double labeling assay showed a significantly decreased bone formation rate in *alpl*^+/−^ mice compared with that of WT and *alpl*^+/−^ mice treated with ionomycin. Scale bar, 25 μm. **e** The intracellular level of Ca^2+^ in *alpl*^+/−^ BMSCs was decreased compared with that in WT BMSCs, and ionomycin treatment elevated the intracellular level of Ca^2+^ in *alpl*^+/−^ BMSCs. **f** Alizarin red staining showed that *alpl*^+/−^ BMSCs cultured under osteoinductive conditions had a decreased capacity to form mineralized nodules compared with that of WT BMSCs. Ionomycin treatment of *alpl*^+/−^ BMSCs showed an elevated capacity to form mineralized nodules. **g** Western blot analysis showed that *alpl*^+/−^ BMSCs had decreased expression of osteogenic-related proteins RUNX2 and Sp7 compared with WT BMSCs and ionomycin treatment of *alpl*^+/−^ BMSCs. β-actin was used as a protein loading control. *alpl*^+/−^ BMSCs showed an increased number of oil red O-positive cells when cultured under adipogenic conditions (**h**), and western blotting showed that there was an upregulation of the adipogenic-related proteins PPARγ2 and LPL (**i**) compared with levels in WT BMSCs and *alpl*^+/−^ BMSCs treated with ionomycin. The representative results from three independent experiments are shown. Error bars represent the s.d. from the mean values. *P < 0.05; **P < 0.01; ***P < 0.001

Ionomycin was reported to cause a robust increase in Ca^2+^ influx and an inhibition in calcium channel endocytosis.^25^ Therefore, we treated 12-week-old *alpl*^+/−^ mice with ionomycin intraperitoneally at a dose of 1 mg·kg^−1^ per day for 28 days. We confirmed that ionomycin treatment increased the calcium influx and the membrane expression of Ca_V_1.2 and Ca_V_1.3 in *alpl*^+/−^ BMSCs (Fig. S7a, b). MicroCT and histological analyses showed that bone mineral density (BMD), Bone volume relative to tissue volume (BV/TV), and distal femoral trabecular bone number (Tb.N) in 3-month-old *alpl*^+/−^ mice were markedly decreased compared with that of the control WT littermates (Fig. 6a, b). We treated *alpl*^+/−^ mice with ionomycin, which caused a robust rise in Ca^2+^ influx in cells.^25^ MicroCT and histological analyses showed that BMD, BV/TV, and Tb.N in 3-month-old *alpl*^+/−^ mice treated with ionomycin was markedly increased compared with that in *alpl*^+/−^ mice (Fig. 6a, b). To observe changes in osteogenic/adipogenic lineage differentiation in vivo, we examined the number of adipocytes in the bone marrow of WT, *alpl*^+/−^ mice, and *alpl*^+/−^ mice treated with ionomycin. Interestingly, oil red O staining showed that the number of adipocytes in *alpl*^+/−^ mouse bone marrow was markedly increased compared with that of WT littermates (Fig. 6c), indicating that *alpl* deficiency increased adipogenic lineage differentiation. However, the number of adipocytes in *alpl*^+/−^ mouse bone marrow after ionomycin treatment was markedly decreased compared with that of *alpl*^+/−^ mice (Fig. 6c). To confirm that *alpl* deficiency directly contributes to decreased osteogenesis, a calcein double labeling analysis was used to show a decreased bone formation rate in *alpl*^+/−^ mice and an elevated bone formation rate in *alpl*^+/−^ mice treated with ionomycin (Fig. 6d).

Moreover, we found that the intracellular level of Ca^2+^ in *alpl*^+/−^ BMSCs was decreased compared with that of WT BMSCs, and ionomycin treatment elevated the intracellular level of Ca^2+^ in *alpl*^+/−^ BMSCs (Fig. 6e). We observed impaired osteogenic differentiation and increased adipogenic differentiation in *alpl*^+/−^ BMSCs, as evidenced by decreased mineralized nodule formation and an elevated numbers of adipocytes (Fig. 6f, h). The decreased expression of the osteogenic markers RUNX2 and Sp7 and increased expression of the adipogenic regulators PPARγ2 and LPL were shown by western blotting (Fig. 6g, i). Ionomycin treatment increased the osteogenic differentiation and decreased adipogenic differentiation of *alpl*^+/−^ BMSCs (Fig. 6f–i).

### Ionomycin treatment rescued osteoporosis in BMSC-specific conditional *alpl* knockout mice

**Fig. 7:**
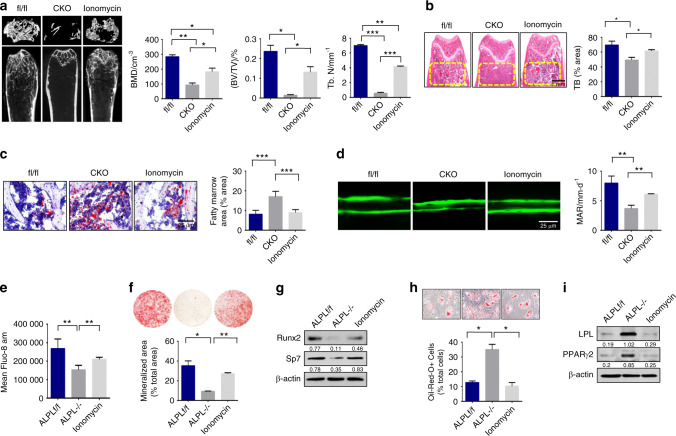
Ionomycin treatment rescued osteoporosis in BMSC-specific conditional *alpl* knockout mice. **a** MicroCT analysis showed that *Prrx1-alpl*^−/−^ mice (*n* = 12) had significantly decreased bone mineral density (BMD), Bone volume relative to tissue volume (BV/TV), and trabecular number (Tb.N) in trabecular bone (TB) of the distal femur compared with the values of their *alpl*^*f/f*^ littermates and *Prrx1-alpl*^−/−^ mice treated with ionomycin (*n* = 10). **b** H&E staining showed a decreased TB volume (yellow circled area) in the distal femurs of *Prrx1-alpl*^−/−^ mice compared with that of the *alpl*^*f/f*^ control group and *Prrx1-alpl*^−/−^ mice treated with ionomycin. Scale bar, 1 mm. **c** Representative histological images of distal femurs show a significantly increased number of adipocytes in *Prrx1-alpl*^−/−^ mouse bone marrow, as assessed by oil red O staining. Scale bar, 25 μm. **d** Calcein double labeling assay showed a significantly decreased bone formation rate in *Prrx1-alpl*^−/−^ mice compared with that of the *alpl*^*f/f*^ controls and *Prrx1-alpl*^−/−^ mice treated with ionomycin. Scale bar, 25 μm. **e** The intracellular level of Ca^2+^ in *alpl*^−/−^ BMSCs was decreased compared with that of the WT BMSCs, and ionomycin treatment elevated the intracellular level of Ca^2+^ in *alpl*^−/−^ BMSCs. **f** Alizarin red staining showed that *alpl*^−/−^ BMSCs had a decreased capacity to form mineralized nodules when compared with the capacity of WT BMSCs cultured under osteoinductive conditions. BMSCs from *Prrx1-alpl*^−/−^ mice treated with ionomycin showed an elevated capacity to form mineralized nodules. **g** Western blot analysis showed that *alp*^−/−^ BMSCs had decreased expression of the osteogenic-related proteins RUNX2 and Sp7 compared with what was observed in WT BMSCs and *alpl*^−/−^ BMSCs treated with ionomycin. β-actin was used as a protein loading control. *alpl*^−/−^ BMSCs showed an increased number of oil red O-positive cells when cultured under adipogenic conditions (**h**), and western blotting showed an upregulation of the adipogenic-related proteins PPARγ2 and LPL (**i**) compared with what was observed in WT BMSCs and *alpl*^−/−^ BMSCs treated with ionomycin. The representative results from three independent experiments are shown. Error bars represent the s.d. from the mean values. *P < 0.05; **P < 0.01; ***P < 0.001

To further determine whether ALPL deficiency in BMSCs caused altered osteogenesis and adipogenesis in vivo, we assessed BV/TV and Tb.N in 3-month-old *Prrx1-alpl*^−/−^ mice, and we found that they were markedly decreased compared with that of their control *alpl*^*f/f*^ littermates (Fig. 7a, b). Floxed *alpl* littermates (*alpl*^*f/f*^) were used as controls. MicroCT and histological analyses showed that, BMD, BV/TV, and Tb.N in 3-month-old *Prrx1-alpl*^−/−^ mice treated with ionomycin were markedly increased compared with what was observed in the *Prrx1-alpl*^−/−^ mice (Fig. 7a, b). To further detect changes in osteogenic/adipogenic lineage differentiation in BMSCs, we examined the number of adipocytes in the bone marrow of *alpl*^*f/f*^, *Prrx1-alpl*^−/−^ mice, and *Prrx1-alpl*^−/−^ mice treated with ionomycin. Oil red O staining showed that the number of adipocytes in *Prrx1-alpl*^−/−^ bone marrow was markedly increased compared with that in the control *alpl*^*f/f*^ littermates (Fig. 7c). However, the number of adipocytes in *Prrx1-alpl*^−/−^ bone marrow after ionomycin treatment was markedly decreased compared with that of the *Prrx1-alpl*^−/−^ mice (Fig. 7c). Calcein double labeling analysis showed a decreased bone formation rate in *Prrx1-alpl*^−/−^ mice relative to that of control *alpl*^*f/f*^ mice (Fig. 7d). Ionomycin treatment reversed the impaired osteogenesis in *Prrx1-alpl*^−/−^ mice. Moreover, the serum levels of RANKL and OPG were not significantly changed, as assessed by ELISA (Fig. S7c, d), suggesting that osteoclasts may not be altered in *Prrx1-alpl*^−/−^ mice. The intracellular level of Ca^2+^ in *alpl*^−/−^ BMSCs was decreased compared with that in the control BMSCs, and ionomycin treatment elevated the intracellular level of Ca^2+^ in *alpl*^−/−^ BMSCs (Fig. 7e). In addition, BMSCs from *Prrx1-alpl*^−/−^ mice showed decreased osteogenic differentiation and increased adipogenic differentiation compared with BMSCs from *alpl*^*f/f*^ mice (Fig. 7f–i). BMSCs from *Prrx1-alpl*^−/−^ mice treated with ionomycin showed increased osteogenic and decreased adipogenic differentiation (Fig. 7f–i). These results indicate that ALPL deficiency in BMSCs induces an age-related osteoporosis phenotype and that ionomycin treatment reversed this phenotype.

### ALPL deficiency promoted the internalization of L-type Ca^2+^ channels in HPP patient-derived BMSCs

**Fig. 8:**
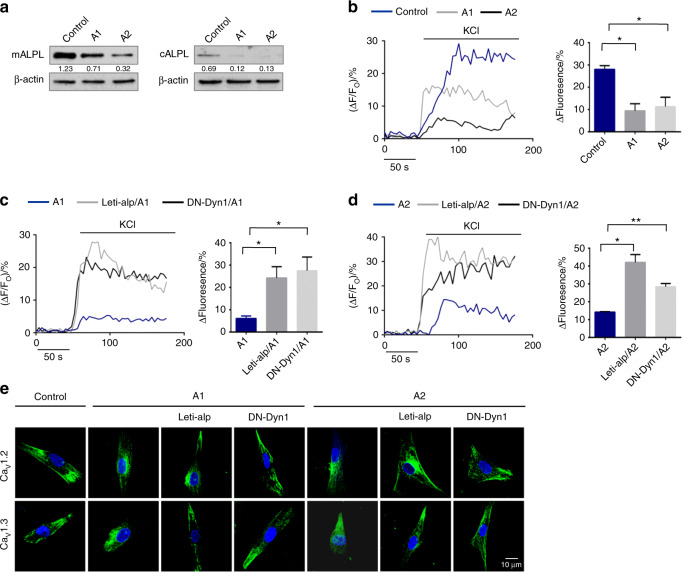
ALPL deficiency promoted the internalization of L-type Ca^2+^ channels in HPP patient-derived BMSCs. **a** The expression of ALPL on the membrane and cytoplasm was decreased in BMSCs from two HPP patients compared with that of normal human BMSCs. β-actin was used as a protein loading control. **b** Intracellular Ca^2+^ imaging analysis showed that KCl-induced Ca^2+^ influx was significantly decreased in cultured BMSCs from HPP patients compared with that of normal human BMSCs. **c** Overexpression of ALPL or transfection with DN-Dyn1 in BMSCs from A1 patients showed elevated KCl-induced Ca^2+^ influx. **d** Overexpression of ALPL or transfection with DN-Dyn1 in BMSCs from A2 patients showed elevated KCl-induced Ca^2+^ influx. **e** Representative images of confocal laser scanning microscopy showing the membrane location of Ca_V_1.2 and Ca_V_1.3 in control BMSCs, A1 BMSCs, A2 BMSCs, A1 and A2 BMSCs overexpressing ALPL, and A1 and A2 BMSCs transfected with DN-Dyn1. Scale bar, 10 μm. The representative results from three independent experiments are shown. Error bars represent the s.d. from the mean values. *P < 0.05; **P < 0.01

We also collected bone marrow BMSCs from two HPP patients with mutations in the ALPL gene (A1 and A2, Table S1). The expression of ALPL on the membrane and cytoplasm was decreased in BMSCs from the two HPP patients compared with normal human bone marrow BMSCs (control) (Fig. 8a). We further determined whether the lack of ALPL resulted in decreased KCl-induced Ca^2+^ influx in A1 and A2 BMSCs. KCl-induced Ca^2+^ influx was significantly decreased in culture-expanded A1 and A2 BMSCs compared with that of control BMSCs (Fig. 8b), and overexpression of ALPL elevated KCl-induced Ca^2+^ influx (Fig. 8c, d) in A1 and A2 BMSCs. Moreover, we also found that the expression of DN-Dyn1 prevented the decrease in KCl-induced Ca^2+^ influx in A1 and A2 BMSCs (Fig. 8c, d). The overexpression of ALPL or expression of DN-Dyn1 in A1 and A2 BMSCs increased the membrane expression of Ca_V_1.2 and Ca_V_1.3, as shown by confocal images (Fig. 8e), suggesting that ALPL deficiency causes channel internalization of Ca_V_1.2 and Ca_V_1.3 in a human model. To confirm that ALPL regulates the lineage differentiation of BMSCs, we overexpressed ALPL in A1 and A2 BMSCs and assessed their osteogenic or adipogenic ability after induction. A1 and A2 BMSCs transfected with the ALPL vector showed increased osteogenic differentiation and decreased adipogenic differentiation (Fig. S8a–d). All of the above data show that ALPL regulates lineage differentiation of MSCs through association with the α2δ subunit of L-type Ca^2+^ channels and through inhibiting the internalization of L-type Ca^2+^ channels, thus increasing Ca^2+^ influx (Fig. S9).

## Discussion

The deficiencies caused HPP are currently treated by bone anabolic and/or enzyme replacement strategies. Bone anabolic treatment, such as treatment with recombinant human parathyroid hormone analogs yielded debatable efficacy.^26^ Asfotase alfa (Strensiq, Alexion), a bone-targeted enzyme replacement therapy, was approved for the long-term treatment of pediatric-onset HPP in the United States, Europe, Canada, and Japan. However, there are no guidelines for selecting adult patients for treatment, evaluating the results of treatment, or determining the optimal duration of treatment at this time. Patients with HPP can also develop secondary osteoporosis, bone marrow edema, and delayed fracture healing or difficulties with implant failure.^26^ Thus, there is an urgent need to identify further bone-targeted treatment options for adult HPP patients.

Our previous study showed that the Alpl deficiency results in premature bone aging characterized by bone mass loss and simultaneous marrow fat gain. Although a pivotal role for ALPL in skeletal matrix mineralization has been established, the mechanism of ALPL regulating BMSC differentiation remains uncertain. Hypercalcemia was found in severely affected infants with HPP, which suggests that ALPL may modulate calcium homeostasis. In this study, we found that raising the intracellular level of calcium in BMSCs by ionomycin rescued ALPL deficiency-induced age-related osteoporosis, which suggests that targeting calcium channels is a new approach for adult HPP treatment. Moreover, our study showed a new function for ALPL in controlling Ca^2+^ influx by regulating the internalization of calcium channels, which balanced the osteogenic and adipogenic differentiation of BMSCs.

Voltage-dependent calcium channels are an important route by which Ca^2+^ enters cells upon membrane depolarization to regulate calcium homeostasis. A previous study showed that VGCCs in BMSCs and osteoblasts regulate bone formation and that manipulating VGCCs promotes bone repair.^27,28^ Of note, the L-VGCC, a major channel of calcium influx, is a part of the high-voltage activated family of VGCCs. L-type calcium channels are considered to play an important role in regulating BMSC function.^15,16^ L-VGCCs, which consist of four subunits (Ca_V_1.1, Ca_V_1.2, Ca_V_1.3, and Ca_V_1.4), are expressed in a tissue-specific fashion.^29^ Ca_V_1.2 and Ca_V_1.3 constitute the major fraction of L-type calcium channels in mammals.^30–32^ However, how Ca_V_1.2 and Ca_V_1.3 are regulated in BMSCs is still unknown. Here, we found that a lack of ALPL caused a decreased number of Ca_V_1.2 and Ca_V_1.3 cells in the membrane and a decrease in Ca^2+^ influx in BMSCs, which led to the aberrant lineage differentiation of BMSCs.

The involvement of ALPL in channel internalization leads to a change in the Ca_V_1.2 and Ca_V_1.3 number at the surface of cells. Several other proteins have been found to regulate the membrane expression of VGCCs, including calmodulin, Akap15, Akap9, and eIF3e, and these associations play an important role in connecting VGCCs and intracellular signaling pathways.^25^ Of these proteins, ALPL is unusual because it is an ectoenzyme that hydrolyzes several substrates. Our study showed a new function for ALPL in in BMSCs; it controls Ca^2+^ influx by regulating the internalization of Ca_V_1.2 and Ca_V_1.3. The results suggest that ectoenzymes on the cell membrane may bind channels in the cell and regulate their trafficking. Precisely how ALPL regulates L-type Ca^2+^ channel trafficking is unclear. Our colocalization data indicate that there is direct contact between ALPL and L-type Ca^2+^ channels. The α2δ subunit of the L-type Ca^2+^ channel is responsible for regulating the trafficking of channels.^23,24^ Our colocalization data indicate that ALPL may bind to the α2δ subunit to regulate L-type Ca^2+^ channel trafficking. A mutation in the α2δ subunit reduced the expression of Ca_V_1.2 and Ca_V_1.3 at the membrane of cells. However, further studies are required to explore the detailed mechanism of L-type Ca^2+^ channel trafficking. Conditional binding of ALPL to Ca_V_1.2 and Ca_V_1.3 suggests that the composition of the Ca_V_1.2 and Ca_V_1.3 protein complex with ALPL may play an important role in regulating channel trafficking. On the other hand, our previous study showed that ALPL deficiency in BMSCs enhanced ATP release and reduced ATP hydrolysis.^3^ ATP, as the energy source used in channel trafficking, may also play a critical role in L-type Ca^2+^ channel internalization in ALPL deficiency conditions.

Skeletal defects in HPP, include rickets, osteomalacia, fractures, bone pain, and various dental defects.^33,34^ To understand the physiological role of ALPL and evaluate the potential treatments, several lines of ALPL knockout mice were generated.^35,36^ Homozygous mice show severe bone disease, but they often die before puberty.^37^ However, here, we found that ALPL deficiency in BMSCs caused decreased osteogenic differentiation and increased adipogenic differentiation. The *alpl*^+/−^ mouse model phenocopies adult patients with HPP, and these mice showed inhibited bone formation but increased adipose tissue in the bone matrix. Moreover, bone formation was inhibited when we generated *alpl* conditional knockout mice of BMSCs, which was consistent with recent reports.^37^ Further, other recent data have shown that increased marrow adipose tissue is correlated with dysfunction of bone and hematopoietic regeneration.^38^ We also found that bone formation was inhibited but adipose tissue was increased in the bone matrix of *alpl* conditional knockout mice, which suggests that ALPL regulates the osteogenic/adipogenic differentiation of BMSCs and causes osteoporosis in patients with HPP.

Our findings regarding the involvement of ALPL in calcium homeostasis revealed a molecular mechanism underlying the BMSC balance between osteogenic and adipogenic differentiation. The change in Ca^2+^ influx in BMSCs following H_2_S exposure regulates osteogenic differentiation through the PCK/Erk/Wnt pathway.^39^ Here, we demonstrated that the Akt/GSK3β/Wnt/β-catenin pathway was downstream of ALPL-mediated regulation of Ca^2+^ influx. The Akt/GSK3β/Wnt/β-catenin pathway further balanced the osteogenic and adipogenic differentiation of BMSCs and bone formation. In our study, we found that ALPL was required to maintain intracellular Ca^2+^ influx by regulating internalization of the L-type Ca^2+^ channel via binding to the α2δ subunits in BMSCs. Altered intracellular Ca^2+^ influx caused by the ALPL deficiency resulted in an osteoporotic phenotype due to downregulated osteogenic differentiation and upregulated adipogenic differentiation in both human and mouse BMSCs. Inhibition of calcium channel internalization by ionomycin increased calcium influx and enhanced bone formation in *alpl*^+/−^ mice and *Prrx1-alpl*^−/−^ mice, suggesting that targeting calcium channel internalization is a potential treatment strategy for adult patients with HPP.

## Materials and methods

### Mice

C57BL/6J and B6.129S7-*Alpl*^*tm1Sor*^/J mice were purchased from Jackson Laboratory (Bar Harbor, ME, USA). We produced mice that were homozygous for ALPL^flox^ using the CRISPR/Cas9 system; an ALPL allele was generated in which the DNA segment includes exons 3 and 4 was flanked by *loxP* sites. By coinjection of Cas9/sgRNAs and the ALPL targeting vector into zygotes, we generated ALPL-floxed heterozygous mice. To generate a tissue-specific Cre-mediated ALPL knockout model, Prrx1-Cre (C57BL/6-Prrx1^*tm1(iCre)*^/Bcgen) (Beijing Biocytogen Co., Ltd Beijing, China) mice were mated with mice heterozygous for the ALPL-floxed allele. The offspring inherited Prrx1-Cre and two ALPL-floxed alleles. Routine mouse genotyping was performed by PCR. The following primer pairs were used for Cre and floxed alleles: Prrx1^iCre^ primer 1: AGCGTTTGGTGTTGATTCGAGC; Prrx1^iCre^ primer 2: AGTCCCGGTGACTCCAGCAG; Prrx1^iCre^ primer 3: TGGTGCACAGTCAGCAGGTTG; ALPL-3′loxP-F: CCTGCTACCTGCTTGCTGGCAATGG; ALPL-3′loxP-R: AGGACACCAAAGACCAGGGACACTA. All animal experiments were performed in accordance with institutionally approved protocols for the use of animal research (University of Fourth Military Medical University protocol number k9-024).

### Isolation of mouse bone marrow mesenchymal stem cells

Bone marrow cells were flushed from the bone cavities of mouse femurs and tibias with 2% heat-inactivated fetal bovine serum (FBS; Hangzhou Sijiqing Biological Engineering Materials Co., Ltd Zhejiang, China) in PBS. A single-cell suspension of all nucleated cells was obtained by passing all bone marrow cells through a 70 μm cell strainer (BD Bioscience, NJ, USA). Single cells (1 × 10^6^) were seeded in 100 mm culture dishes (Corning, NY, USA) and initially incubated for 48 h at 37 °C and 5% CO_2_. To eliminate the nonadherent cells, the cultures were washed with PBS twice on the second day. The attached cells were cultured for 16 days in alpha minimum essential medium (α-MEM, Gibco BRL, Gaithersburg, MD, USA) supplemented with 20% FBS, 2 mmol·L^−1^ l-glutamine (Invitrogen Life Technology, Carlsbad, CA, USA), 55 μmol·L^−1^ 2-mercaptoethanol (Invitrogen), 100 U·mL^−1^ penicillin, and 100 μg·mL^−1^ streptomycin (Invitrogen). To confirm that the cells had mesenchymal stem cell characteristics, we used flow cytometric analysis to show that these BMSCs were positive for Sca-1, CD44, and CD73 and were negative for CD34 and CD45 (BD Biosciences, San Diego, CA, USA).

### Isolation of human bone marrow mesenchymal stem cells

Two HPP patients aged 10 (male) and 2.5 (female) years were treated by the Affiliated Hospital of Fourth Military Medical University for osteodynia and missing teeth. Healthy human BM samples were collected from five teenagers aged 10–13 years (male) who underwent alveolar bone cleft repair by autoilium transplantation. The cells were purified from the BM using a Percoll density gradient centrifugation method, and then they were cultured in α-MEM supplemented with 10% FBS (Gibco BRL), 2 mmol·L^−1^ L-glutamine (Invitrogen), 100 U·mL^−1^ penicillin, and 100 mg·mL^−1^ streptomycin (Invitrogen) at 37 °C in 5% CO_2_.^3^ BMSCs in their third passage were used in experiments.

### Calcium imaging

Calcium imaging was performed using confocal laser microscopy (Zeiss, Oberkochen FV1000, Germany). The intracellular Ca^2+^ level ([Ca^2+^]_*i*_) was determined by Fluo-3 fluorescence intensity, as described previously.^40^ Briefly, BMSCs were cultured in 12-well plates and were incubated with 5 μmol·L^−1^ Fluo-3/AM dye (Invitrogen, Life Technology, Carlsbad, CA, USA) for 30 min in α-MEM at 37 °C. BMSCs were again washed three times with calibrated EGTA/Ca^2+^ solutions. KCl (30 mmol·L^−1^) or TG (20 μmol·L^−1^, Sigma-Aldrich, St. Louis, MO, USA) was added to test which type of calcium channel was affected. Images were collected every 4 s at 2 Hz with excitation at 488 nm and emission at 530 nm. Data are presented as the Fluo-3 fluorescence intensity increase ratio: *R* = Δ*F/F*_0_, where Δ*F* = *F* *–* *F*_0_. *F* is the fluorescence value detected, and *F*_0_ is the minimum fluorescence value.

### Confocal microscopy

Confocal images were acquired with a Zeiss Oberkochen FV1000 confocal laser scanning microscope using a ×60 oil immersion objective. BMSCs were fixed with 3.7% paraformaldehyde in distilled water at 4 °C for 10 min and then were incubated overnight with primary antibodies, which were followed by incubation with secondary antibodies for 1 h. The nuclei were stained with 1 μg·mL^−1^ Hoechst 33342. The plasma membrane was stained with 5 μg·mL^−1^ of the membrane marker CellMask™ Deep Red Plasma Membrane Stain (Thermo Fisher Scientific, MA, USA). Images were acquired using an argon laser (excitation, 488 nm; emission, BP505-530 nm emission filter) for FITC-labeled Ca_V_1.2 or Ca_V_1.3, a UV laser for excitation and a BP385-470 nm emission filter for Hoechst 33342, and a He–Ne laser (excitation, 543 nm; emission filter, LP650 nm) for Cy3-labeled ALPL. BMSCs (1 × 10^5^) were plated onto coverslips, and the next day cells were treated with 10 μmol·L^−1^ ATP, 10 μmol·L^−1^ ppi, or 1 μg·mL^−1^ ionomycin for 1 h before immunofluorescence staining for Ca_V_1.2 or Ca_V_1.3. Plasma membrane localization of Ca_V_1.2 or Ca_V_1.3 in BMSCs, as visualized by staining with anti-Ca_V_1.2 or anti-Ca_V_1.3 antibodies, was recorded for more than ten cells. Colocalization of the L-type Ca^2+^ channel (Ca_V_1.2 or Ca_V_1.3) and ALPL was also observed using a laser scanning confocal microscope, and images were obtained using FV10-ASW Viewer 4.2 (Zeiss, Oberkochen FV1000, Germany).

To record the time-course change of internalization of Ca_V_1.2, a plasmid encoding DsRed-Cav1.2 was generated and transfected into the BMSCs. The plasmid was constructed by introducing a DsRed segment into the Ca_V_1.2 plasmid (Addgene plasmid #26572) according to the instructions of a ClonExpress® II One Step Cloning kit (Vazyme, Nanjing, China). Dio (Thermo Fisher Scientific, MA, USA) was used to label the cell membrane, and DAPI (Thermo Fisher Scientific) was used to label the nucleus. The average colocalization intensity was determined by selecting an ROI corresponding to the cell’s footprint in the first image and measuring the average intensity in that region over the entire time course. The ROI was visually selected in a region of the Dio-labeled plasma membrane. Cells that were outside of this ROI were excluded from analysis. The amount of time channels spent at the membrane was measured for more than 15 ROIs for at least five cells per condition. We recorded the time-dependent red fluorescence intensity of these regions. Quantitative data are presented as the fluorescence intensity increase ratio: *R* = *F/F*_0_. *F* is the fluorescence value detected, and *F*_0_ is the first detected fluorescence value. Quantification of the fluorescence density of ROIs at 0 s, 300 s, and 600 s was analyzed by NIH ImageJ software.

### Cell-surface protein isolation

The expression level of Ca^2+^ channels in the surface membrane was determined by isolating cell-surface proteins using a plasma membrane protein extraction kit (Abcam, Cambridge, UK). In brief, the cells were lysed using homogenization buffer containing protease inhibitor cocktail. The homogenate was centrifuged for 10 min at 700 *g*, and the pellet was discarded. The supernatant was then centrifuged for 30 min at 10 000 *g* and 4 °C to obtain the total cellular membrane protein. Then, purification of the plasma membrane proteins was carried out according to the manufacturer’s protocol. The protein concentrations were measured with a Bradford protein assay kit (Beyotime, Shanghai, China). Then, equal amounts of the cytoplasmic and membrane proteins were saved as direct input for immunoblot experiments.

### Osteogenic differentiation

BMSCs were cultured under osteogenic culture conditions in growth medium containing 2 mmol·L^−1^ β-glycerophosphate (Sigma-Aldrich, St. Louis, MO, USA), 100 μmol·L^−1^ L-ascorbic acid 2-phosphate (MP Biomedicals, Irvine, CA, USA), and 10 nmol·L^−1^ dexamethasone (Sigma-Aldrich, St. Louis, MO, USA). Two weeks after induction, staining was performed with 1% alizarin red S (Sigma-Aldrich) for 3 min at room temperature to detect matrix mineralization. Then, 10 μmol·L^−1^ SC79 or 10 mmol·L^−1^ LicL was added to the osteogenic culture medium for induction. The stained areas were quantified using NIH ImageJ software and are shown as a percentage of the total area.

### Adipogenic differentiation

For adipogenic induction, 500 nmol·L^−1^ isobutylmethylxanthine (MP Biomedicals, Irvine, CA, USA), 60 μmol·L^−1^ indomethacin (Sigma-Aldrich), 500 nmol·L^−1^ hydrocortisone (MP Biomedicals, Irvine, CA, USA), 10 μg·mL^−1^ insulin (Sigma-Aldrich), and 100 nmol·L^−1^ L-ascorbic acid phosphate were added into the growth medium. Then, 10 μmol·L^−1^ SC79 or 10 mmol·L^−1^ LicL was added to the adipogenic culture medium for induction. After 7 days, the cultured cells were stained with oil red O (Sigma-Aldrich), and positive cells were quantified under microscopy and are shown as a percentage of the total cells.

### Western blotting

Cells were lysed using M-PER mammalian protein extraction reagent (Thermo, MA, USA) with protease and phosphatase inhibitors (Roche, Basel, Switzerland), and proteins were quantified using protein assays (Bio-Rad Laboratories, Shanghai, China). Twenty micrograms of protein was separated by SDS-PAGE and then were transferred to nitrocellulose membranes (Millipore, Billerica, MA, USA). Membranes were blocked with 0.1% Tween-20 and 5% BSA for 1 h before overnight incubation with a primary antibody diluted in blocking solution. Antibodies against mouse ALP were purchased from R&D Systems, and antibodies against Ca_V_1.1, Ca_V_1.2, Ca_V_1.3, Ca_V_2.1, Ca_V_2.2, Ca_V_3.1, Ca_V_3.2, Ca_V_3.3, and Ca_V_α2δ1 were purchased from Alomone Labs (Alomone, Jerusalem, Israel). Antibodies against mouse phospho-Erk1/2 (Thr202/Tyr204), Erk1/2, GSK3β, phospho-GSK3β, phospho-AKT, β-catenin, RUNX2, SP7, Bglap, PPARγ2, LPL, phospho-PKC, PKC, phospho-CamkII, and AKT were purchased from Abcam (Cambridge, UK). Antibodies against mouse Ibsp were purchased from Absin (Shanghai, China). Antibodies against mouse CamkII and active β-catenin were obtained from Millipore (Billerica, MA, USA). Antibodies against mouse β-actin were purchased from Boster (Wuhan, China). The membranes were incubated for 1 h in HRP-conjugated secondary antibody diluted at 1:40 000 in blocking solution. Immunoreactive proteins were detected using an enhanced chemiluminescence kit (Amersham Biosciences, Piscataway, NJ, USA). The intensity of bands was measured using NIH ImageJ software, and data were normalized to β-actin.

### Coimmunoprecipitation

To test whether ALPL and L-VGCC (Ca_V_1.2 or Ca_V_1.3) interact in cells, coimmunoprecipitation was performed as previously described.^41^ The cells were completely lysed in cell lysis buffer for western blotting and IP (Beyotime, Shanghai, China). The lysates were incubated with primary antibodies overnight at 4 °C, and then protein A/G magnetic beads (Millipore, USA) were added for 2 h at 4 °C. Immunocomplexes were washed three times with PBS containing 0.1% Tween-20 and were subsequently subjected to western blot analysis.

### Biotinylation assays

A surface biotinylation assay was performed following the manufacturers’ protocols for EZ-link Sulfo-NHS-LC-LC-biotin and Immobilized/NeutrAvidin Ultralink Resin (Thermo Fisher Scientific, MA, USA). For the surface biotinylation assay, the cells were incubated with 1 mg·mL^−1^ EZ-linked Sulfo-NHS-LC-LC-biotin in DPBS for 30 min at room temperature, and unreacted biotin was quenched with cold 100 mmol·L^−1^ glycine in DPBS. Then, we used neutravidin ultralink resin beads to capture biotinylated surface proteins. The beads were washed three times with lysis buffer (50 mmol·L^−1^ Tris/HCl [pH 7.4], 1 mmol·L^−1^ EDTA, 150 mmol·L^−1^ NaCl, and protease inhibitors), and then elution was performed with loading buffer. After SDS-PAGE, western blot analysis of the captured proteins was performed with anti-Ca_V_1.2 and anti-Ca_V_1.3 antibodies (Alomone, Jerusalem, Israel).

### Plasmid construction

For shRNA knockdown experiments, we constructed an shALPL lentiviral vector according to the protocol of the pLKO.1-TRC cloning vector.^42^ In brief, to generate oligos for cloning into pLKO.1, we synthesized the following oligonucleotides: forward oligo: CCGGGCAGTATGAATTGAATCGGAACTCGAGTTCCGATTCAATTCATACTGCTTTTTG, reverse oligo: AATTCAAAAAGCAGTATGAATTGAATCGGAACTCGAGTTCCGATTCAATTCATACTGC.

Then, the forward and reverse oligos were annealed and ligated into the pLKO.1 vector, producing a final plasmid that expressed an shRNA targeting ALPL. To overexpress ALPL in BMSCs, we constructed a lentiviral vector with ALPL cDNA. The ALPL (GenBank Accession No. NM_000478.5) cDNA was amplified by PCR using the primer pairs: forward: 5′-ACTGGATCCTCCAGGGATAAAGCAGGTCT-3′; reverse: 5′- TATCTCGAGTGGGAAGTTGGCATCTGTC-3′. Then, the ALPL gene was inserted into the vector pENTR^TM^2B (Invitrogen Life Technology, Carlsbad, CA, USA), and a Gateway LR recombination reaction between the ALPL clone vector and pLenti6.3/V5-DEST was performed to generate the expression clone Lenti-ALPL. The cDNA encoding β-catenin (GenBank Accession No. NM_007614.3) was amplified by TaKaRa LA Taq polymerase with GC Buffer (TaKaRa, Japan). Primers were used as follows: forward: 5′-CGGGGAGGCGGAGACGGAGCAC-3′, reverse: 5′-CCAGCCCACCCCTCGAGCCCTCTC-3′. Then, the restriction enzymes SalI and BamHI were used to introduce the above fragment into the backbone vector pIRES2-EGFP (Clonetech, USA).

pDsRed-Cav1.2 was constructed using a ClonExpress Ultra One Step Cloning kit according to the manufacturer’s protocol (Vazyme, Nanjing, China). In brief, Cav1.2 was amplified by primers: forward: 5′-GGGGTACCATGGTCAATGAAAACACGAGG-3′, reverse: 5′-ATAAGAATGCGGCCGC CTACAGGTTGCTGACGTAGGAC-3′ from pCa_V_1.2 (Addgene, Plasmid #26572). Then, the fragment was introduced into the vector pCa_V_1.2 by restriction enzymes KpnI and NotI to construct the recombinant plasmid pCa_V_1.2-KN. DsRed was amplified from pLVX-EF1α-DsRed-monomer-N1 (Biovector NTCC Inc., Beijing, China) with the following primers: forward 5′-GGGAGACCCAAGCTGGCTAGC ATGGACAACACCGAGGACGTCAT-3′, and reverse: 5′-GTTTTCATTGACCATGGTACC CTGGGAGCCGGAGTGGCG-3′. Finally, the DsRed fragment was ligated with linearized pCa_V_1.2-KN by ExnaseII to construct pDsRed-Cav1.2.

### Transfection

For transfecting experiments, BMSCs (0.5 × 10^6^) were seeded on a six-well culture plate and then were transfected with Ca_V_1.2 siRNA or Ca_V_1.3 siRNA (Santa Cruz, Dallas, TX, USA) using X-tremeGENE siRNA Transfection Reagent (Roche, Basel, Switzerland) according to the manufacturer’s instructions.

To downregulate or overexpress ALPL in BMSCs, we first produced lentiviruses carrying shALPL or ALPL cDNA. The lentiviral vector and the ViraPower Packaging Mix were cotransfected into 293T cells to produce a lentiviral stock according to the protocol provided by the manufacturer. Virus-containing supernatants were harvested 48 h after transfection, and then they were pooled and filtered through 0.45-µm filters. Cells were treated with a lentivirus at a multiplicity of infection of 100 at 37 °C and 5% CO_2_. The plates were swirled every 15 min, and fresh medium was added after 1 h of incubation. The cells were exposed to lentivirus for 48 h, which was followed by protein extraction for western immunoblotting or differentiation induction.

The following plasmids were used in transfection experiments: pCatenin-EGFP, pCa_V_1.2 (Addgene, Plasmid #26572), pCa_V_1.3 (Addgene, Plasmid #49332), DN-Dyn1 (Addgene, Plasmid #55795), pCa_V_α2δ1 (Addgene, Plasmid #26575), and pCa_V_α2δ1mut (Addgene, Plasmid #58730). Transfections were performed according to the protocol of X-tremeGENE HP DNA Transfection Reagent (Roche, Basel, Switzerland). After transfection, protein was extracted from cells for western immunoblotting, differentiation induction, and confocal imaging.

### MicroCT analysis

Femurs were harvested and analyzed using a desktop microCT system (eXplore Locus SP, GE Healthcare, USA). The scanner was set at a voltage of 80 kVp, a current of 80 μA and a resolution of 8 μm per pixel. Cross-sectional images of middiaphysis femurs were used to perform three-dimensional histomorphometric analysis of trabecular bone. Cross-sectional volumetric BMD was measured for the right femur middiaphysis with a density phantom. Using three-dimensional images, an ROI in the secondary spongiosa was manually drawn near the endocortical surface. Bone volume relative to tissue volume (BV/TV) and Tb.N were assessed as cancellous bone morphometric parameters.

### Histology

To assess the areas of trabecular bone and bone marrow, femurs and tibias were fixed in 4% paraformaldehyde (Sigma-Aldrich, St. Louis, MO, USA) and then were decalcified with 5% EDTA (pH 7.4), which was followed by paraffin embedding. The 6 μm paraffin sections were stained with hematoxylin and eosin (H&E) and were analyzed using NIH ImageJ software. To label the matrix mineralization, the mice were given intraperitoneal injections of calcein (Sigma-Aldrich, USA, 20 mg·kg^−1^ body weight) at day 10 and day 3 before sacrifice. Bone dynamic histomorphometric analyses for MAR were performed according to the standardized nomenclature for bone histomorphometry using fluorescence microscopy (Leica DM 6000B, German).

### In vivo oil red O staining

To assess adipose tissue in trabecular areas, femurs were fixed in 4% paraformaldehyde and were decalcified with 5% EDTA (pH 7.4), which was followed by cryosectioning. Sections were stained with oil red O, and positive areas were quantified under microscopy and are shown as a percentage of the total area. Briefly, sections were washed with 60% isopropanol and then were incubated in fresh oil red O staining solution for 15 min at room temperature before being counterstained with hematoxylin. All reagents for oil red O staining were purchased from Sigma-Aldrich.

### Ionomycin treatment

Ionomycin (Alomone, Jerusalem, Israel) was dissolved in DMSO. For in vivo treatment, ionomycin was intraperitoneally administered to 12-week-old *alpl*^+/−^ mice and *alpl*^−/−^ CKO mice at a dose of 1 mg·kg^−1^ per day for 28 days. The control mice were treated with only the vehicle. After ionomycin treatment, all groups of mice were healthy.

### Statistics

All experimental group sizes were chosen to ensure adequate statistical power despite the highly variable nature of the studies performed. No animals were excluded, and animals were randomly assigned groups for the studies. Experiments were not performed in a blinded fashion. Data were assessed for normal distribution and similar variance between groups. Comparisons between two groups were performed using independent unpaired two-tailed Student’s *t* tests, and comparisons between more than two groups were analyzed using one-way ANOVA with the Bonferroni adjustment. *P* values of less than 0.05 were considered statistically significant.

## Supplementary information

supplementary data
