# Supplementary material for: Ionomycin ameliorates hypophosphatasia via rescuing alkaline phosphatase deficiency-mediated L-type Ca2+ channel internalization in mesenchymal stem cells
Source: Bone Res. 2020 Apr 26;8:19. doi: 10.1038/s41413-020-0090-7 (PMC7183511; doi:10.1038/s41413-020-0090-7)
Supplement: Supplementary file 1 — supplementary data [file 41413_2020_90_MOESM1_ESM.pdf]

Ionomycin Ameliorates Hypophosphatasia via Rescuing Alkaline Phosphatase Deficiency-mediated L-type Ca2+ Channel Internalization in Mesenchymal Stem Cells

Bei Li[1, 2, 7], Xiaoning He[1, 2, 7], Zhiwei Dong[1, 7], Kun Xuan[5], Wei Sun[6], Li Gao[5], Shiyu Liu[1, 2], Wenjia Liu[1, 2], Chenghu Hu[1, 2], Yimin Zhao[1], Songtao Shi[3, 4] *, Yan Jin[1, 2] *.

Fig. S1. ALPL deficiency caused decreased membrane expression of L-type Ca^{2+} channel in BMSCs.

Fig. S2. ALPL deficiency caused decreased membrane expression of L-type Ca^{2+} channel in BMSCs.

Fig. S3. ALPL maintained BMSC osteogenic/adipogenic lineage differentiation via L-type Ca^{2+} channel.

Fig. S4. ALPL deficiency promoted the internalization of L-type Ca^{2+} channel in BMSCs.

Fig. S5. ALPL deficiency promoted the internalization of L-type Ca^{2+} channel *via* binding to α2δ subunits.

Fig. S6. ALPL deficiency caused aberrant lineage differentiation of BMSCs through Wnt/β-catenin pathway.

Fig. S7. Targeting channel internalization rescued ALPL deficiency caused osteoporosis of HPP.

Fig. S8. ALPL deficiency promoted the internalization of L-type Ca^{2+} channel in HPP patient derived BMSCs.

Fig. S9. Graphic Summary shows the mechanisms of ALPL in regulating MSC lineage commitment.

Table S1. Baseline table of two HPP patients.

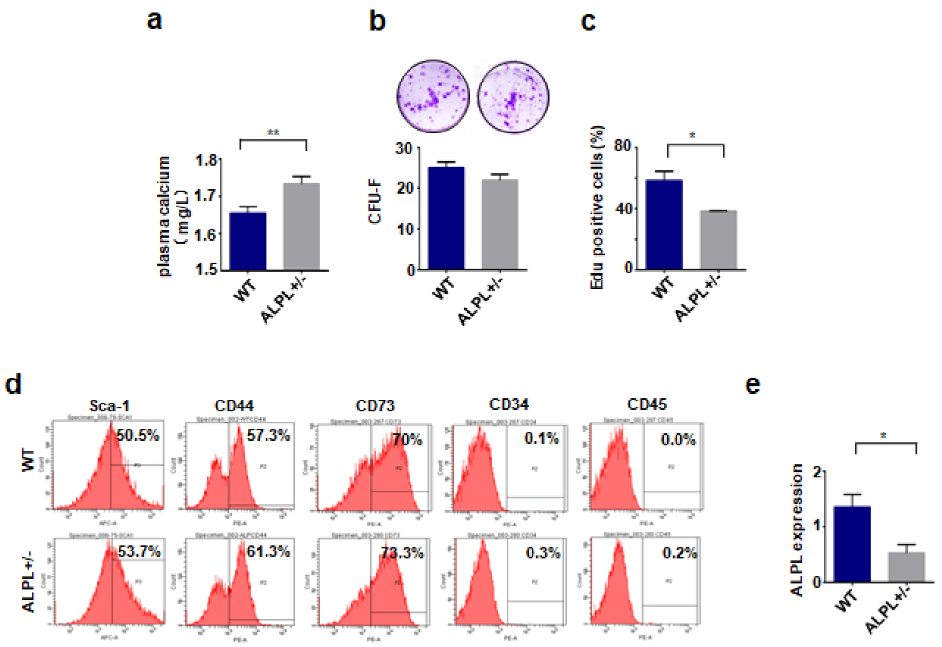

Figure S1. ALPL deficiency caused decreased membrane expression of L-type Ca^{2+} channel in BMSCs. (a) Plasma calcium of WT and *alpl*$^{+/-}$ mice (n=8). Plasma calcium was elevated in *alpl*$^{+/-}$ mice. (b) CFU-F of WT and *alpl*$^{+/-}$ BMSCs. (c) Proliferation of WT and *alpl*$^{+/-}$ BMSCs. *alpl*$^{+/-}$ BMSCs showed decreased rate of Edu positive cells compared to WT BMSCs. (d) FACS analysis of WT and *alpl*$^{+/-}$ BMSCs. (e) Real-time PCR showed decreased expression of ALPL in *alpl*$^{+/-}$ BMSCs compared to WT BMSCs. Representative results were from three independent experiments. *P < 0.05, **P < 0.01.

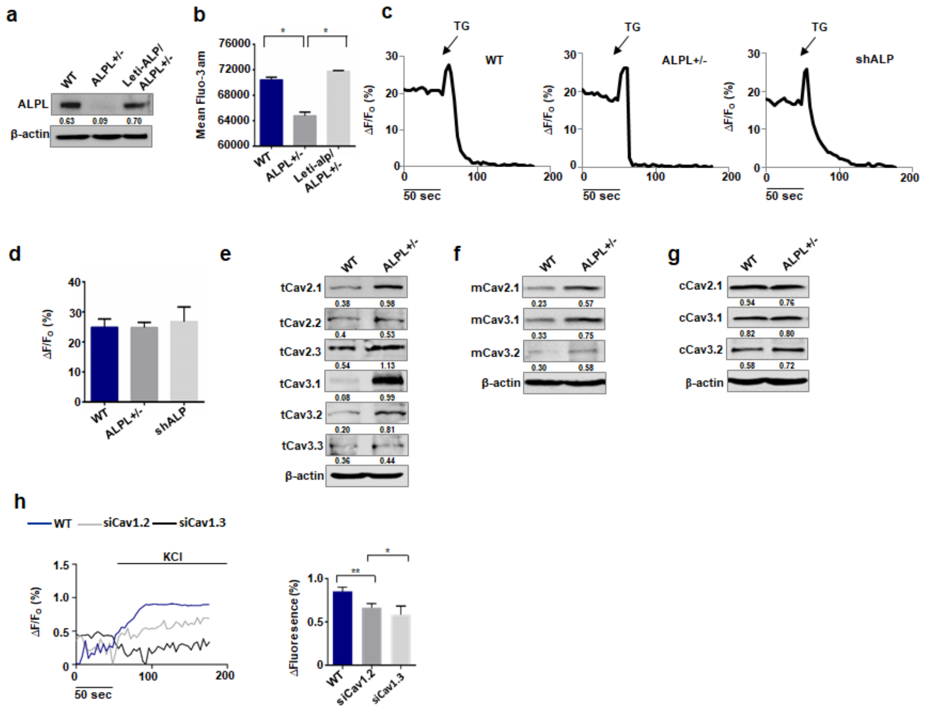

Figure S2. ALPL deficiency caused decreased membrane expression of L-type Ca^{2+} channel in BMSCs. (a) ALPL expression of *alpl*$^{+/-}$ BMSCs was increased after lentiviral transduction with lentivirus-overexpressing ALPL. (b) Cytosolic Ca^{2+} of WT, *alpl*$^{+/-}$ BMSCs and *alpl*$^{+/-}$ BMSCs overexpressed with ALPL. (c, d) 10 nM TG-induced intracellular Ca^{2+} influx was detected of WT BMSCs, *alpl*$^{+/-}$ BMSCs and shALP (n=10). No significant difference of TG-induced intracellular Ca^{2+} influx was found. (e) Total protein expressions of Ca_V2.1, Ca_V2.2, Ca_V2.3, Ca_V3.1, Ca_V3.2 and Ca_V3.3 in WT and *alpl*$^{+/-}$ BMSCs. (f) Membrane expressions of Ca_V2.1, Ca_V3.1, Ca_V3.2 in WT and *alpl*$^{+/-}$ BMSCs. (g) Cytoplasm expressions of Ca_V2.1, Ca_V3.1, Ca_V3.2 in WT and *alpl*$^{+/-}$ BMSCs. (h) Knockdown of Ca_V1.2 and Ca_V1.3 by siRNA resulted in decreased Ca^{2+} influx in WT BMSCs when stimulated with 30 mM KCl for 3 min (n=10). Representative results were from three independent experiments. *$P < 0.05$, ** $P < 0.01$.

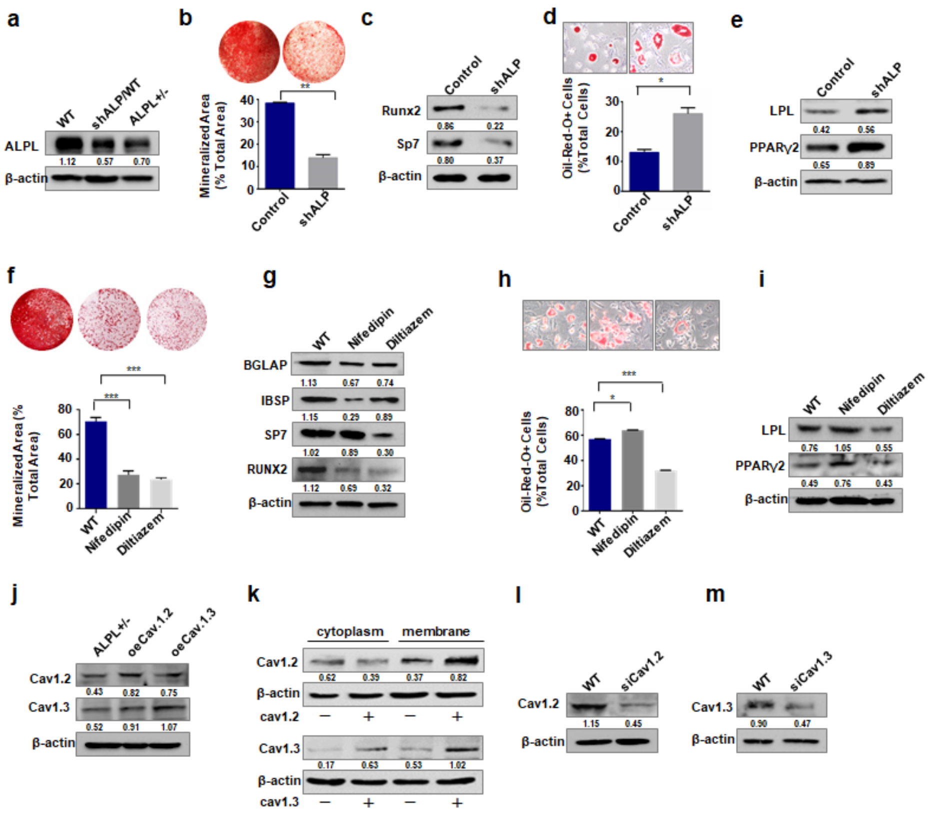

Figure S3. ALPL maintained BMSC osteogenic/adipogenic lineage differentiation via L-type Ca^{2+} channel. (a) ALPL expression of WT BMSCs was decreased after transfected with shALP. (b, c) Alizarin red staining showed shALP BMSCs had a decreased capacity to form mineralized nodules when cultured under osteo-inductive conditions (b). Western blot analysis showed shALP BMSCs had decreased expression of osteogenic related proteins RUNX2 and Sp7 (c). (d, e) shALP BMSCs showed an increased number of Oil red O-positive cells when cultured under adipo-inductive conditions (d) and upregulation of the adipogenic related proteins PPARγ2 and LPL, as assessed by western blot (e). (f, g) Diltiazem or nifedipine treatment reduced osteogenic differentiation of WT BMSCs as assessed by alizarin red staining to show mineralized nodule formation (f) and western blot to show the expression levels of BGLAP, IBSP, RUNX2 and Sp7 (g). (h, i) Nifedipine treatment elevated adipogenic differentiation of WT BMSCs as assessed by Oil red O-staining to show the number of adipocytes and western blot to show the expression levels of PPARγ2 and LPL (h). In contrast, diltiazem treatment reduced adipogenic differentiation of WT BMSCs as assessed by Oil red O-staining to show the number of adipocytes and western blot to show the expression levels of PPARγ2 and LPL (i). (j) The expression of Ca$_V$1.2 or Ca$_V$1.3 in *alpl*$^{+/-}$ BMSCs was increased after transfection with overexpressing

plasmid $Ca_V1.2$ or $Ca_V1.3$. (k) Plasmid-overexpressed $Ca_V1.2$ and $Ca_V1.3$ of *alpl*$^{+/-}$ BMSCs showed upregulated membrane expression of both channels by western blot analysis. (l) $Ca_V1.2$ expression of WT BMSCs was decreased after transfection with $Ca_V1.2$ siRNA. (m) $Ca_V1.3$ expression of WT BMSCs was decreased after transfection with $Ca_V1.3$ siRNA. β-actin was used as a protein loading control. Representative results were from three independent experiments. *P < 0.05, **P < 0.01, ***P < 0.001.

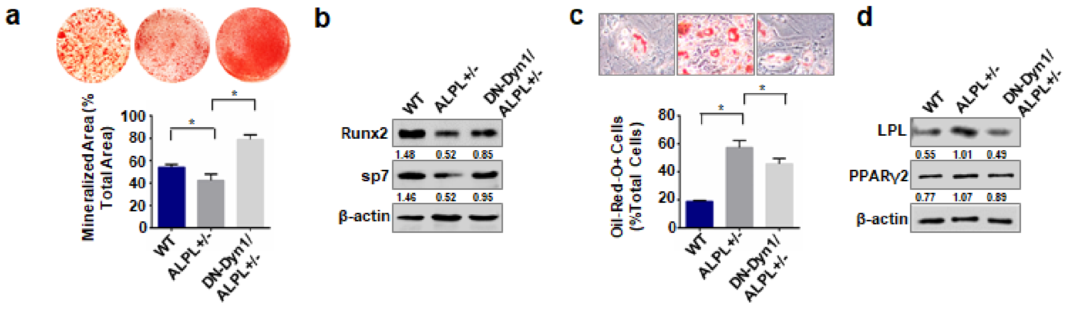

Figure S4. ALPL deficiency promoted the internalization of L-type Ca^{2+} channel in BMSCs. (a, b) Alizarin red staining showed that *alpl*$^{+/-}$ BMSCs transfected with DN-Dyn1 had an increased capacity to form mineralized nodules when cultured under osteo-inductive conditions (a) and upregulation of the osteogenic related proteins RUNX2 and Sp7 (b). (c, d) *alpl*$^{+/-}$ BMSCs transfected with DN-Dyn1 showed a decreased number of Oil red O-positive adipocytes when cultured under adipo-inductive conditions (c) and downregulation of the adipogenic related proteins PPARγ2 and LPL, as assessed by western blot (d). Representative results were from three independent experiments. *P<0.05.

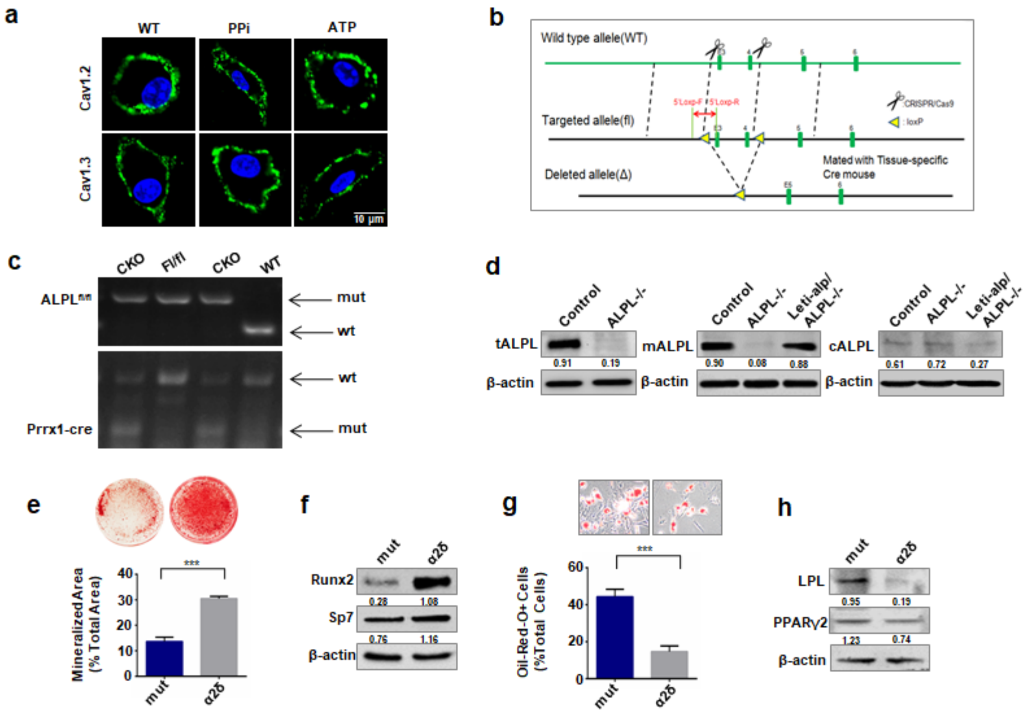

Figure S5. ALPL deficiency promoted the internalization of L-type Ca^{2+} channel *via* binding to α2δ subunits. (a) Representative images of confocal laser scanning microscope showed membrane location of Ca_V1.2 and Ca_V1.3 in BMSCs and BMSCs treated by PPi or ATP. Scale bar, 10 μm. (b) Targeting strategy for conditional knockout of the gene for ALPL. Structure of the genomic ALPL locus, targeting vector, and the homologous recombined allele were shown. Exons are depicted as green boxes, and intronic sequences are shown as solid lines. Forward and reverse primers used for PCR genotyping for floxed alleles were shown as red arrowheads. (c) PCR genotyping of Cre transgenes and floxed alleles. (d) Expression of total ALPL, membrane ALPL and cytoplasm ALPL in BMSCs from *alpl*$^{f/f}$ mice and *Prrx1-alpl*$^{-/-}$ mice. After overexpression of ALPL in BMSCs from *Prrx1-alpl*$^{-/-}$ mice , the expression of membrane ALPL was increased. (e, f) Alizarin red staining showed that BMSCs transfected with mutant $\alpha_2\delta$ had a decreased capacity to form mineralized nodules compared to BMSCs transfected with $\alpha_2\delta$ when cultured under osteo-inductive conditions (e). Western

blot analysis showed BMSCs transfected with mutant $\alpha_2\delta$ had decreased expression of osteogenic related proteins RUNX2 and Sp7 compared to BMSCs transfected with $\alpha_2\delta$ (f). (g, h) BMSCs transfected with mutant $\alpha_2\delta$ showed an increased number of Oil red O-positive cells compared to BMSCs transfected with $\alpha_2\delta$ when cultured under adipo-inductive conditions (g) and upregulation of the adipogenic related proteins PPARγ2 and LPL, as assessed by western blot (h). β-actin was used as a protein loading control. Representative results were from three independent experiments. $^{***}P < 0.001$

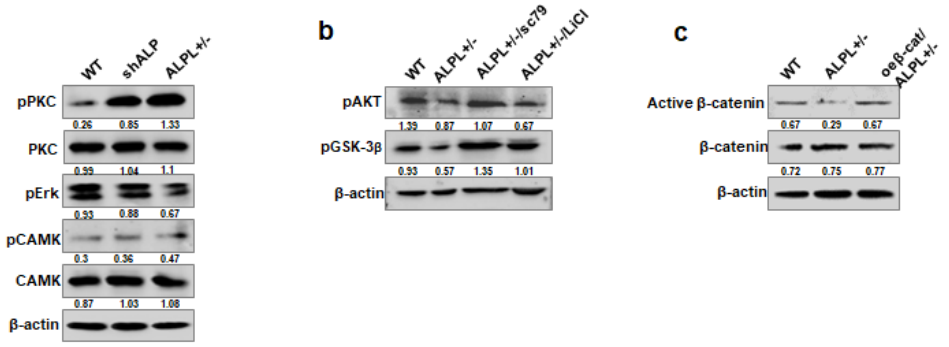

Figure S6. ALPL deficiency caused aberrant lineage differentiation of BMSCs through Wnt/β-catenin Pathway. (a) The expression of pPKC, PKC, pErk, pCAMK, CAMK in WT, $alpl^{+/-}$ BMSCs and shALP BMSCs. The expression of pPKC was increased in $alpl^{+/-}$ BMSCs and shALP BMSCs. However, pErk expression was not changed in $alpl^{+/-}$ BMSCs and shALP BMSCs compared to WT BMSCs. The expression of pCAMK and CAMK was not changed significantly in WT, $alpl^{+/-}$ BMSCs and shALP BMSCs. (b) The expression of pAKT, pGSK-3β in WT, $alpl^{+/-}$ BMSCs and $alpl^{+/-}$ BMSCs treated with sc79 or LiCl. The expressions of pAKT and pGSK-3β were increased in $alpl^{+/-}$ BMSCs after treatment by sc79, but only pGSK-3β expression in $alpl^{+/-}$ BMSCs was increased after treatment by LiCl. (c) The expression of active β-catenin and β-catenin in WT, $alpl^{+/-}$ BMSCs and $alpl^{+/-}$ BMSCs overexpressed β-catenin. The expression of active β-catenin was decreased in $alpl^{+/-}$ BMSCs. After overexpression of β-catenin, the expression of active β-catenin was increased to the

same level of WT BMSCs. β-actin was used as a protein loading control. Representative results were from three independent experiments.

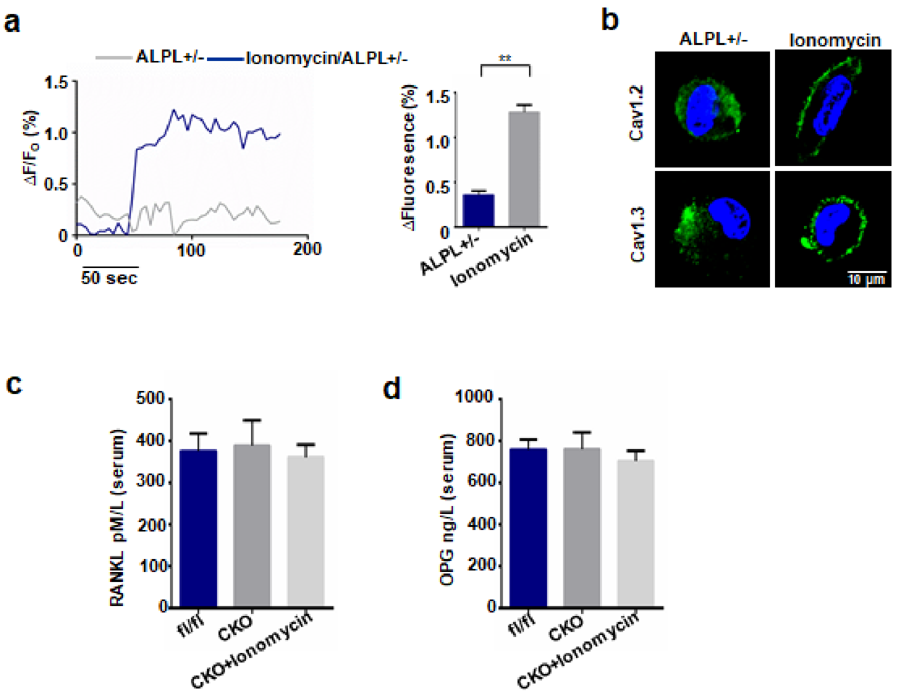

Figure S7. Targeting channel internalization rescued ALPL deficiency caused osteoporosis of HPP.

(a) Ca^{2+} imaging showed increased Ca^{2+} influx of cultured *alpl*$^{+/-}$ BMSCs after treated by ionomycin stimulated with 30 mM KCl for 3 min (n=10). (b) Representative images of confocal laser scanning microscope showed membrane location of Ca$_V$1.2 and Ca$_V$1.3 in cultured *alpl*$^{+/-}$ BMSCs after treated by ionomycin. (c, d) RANKL and OPG expression in serum were measured in WT, *Prrx1-alpl*$^{-/-}$ mice and *Prrx1-alpl*$^{-/-}$ mice treated by ionomycin. The serum levels of RANKL and OPG were not significantly changed, as assessed by ELISA. Representative results were from three independent experiments. ** $P < 0.01$.

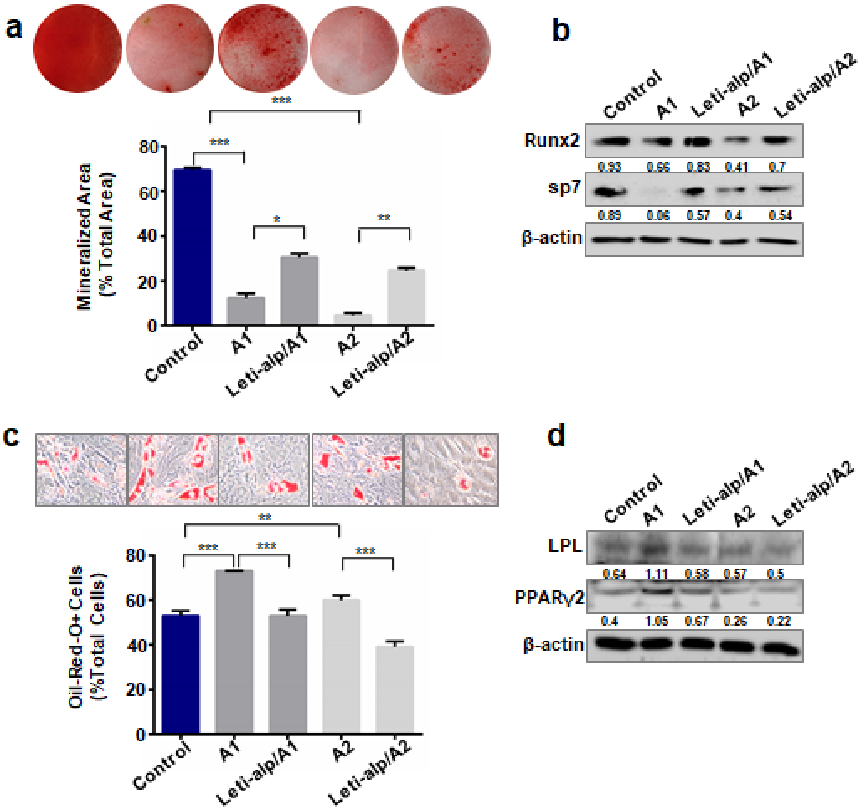

Figure S8. ALPL deficiency promoted the internalization of L-type Ca^{2+} channel in HPP patient derived BMSCs. (a, b) Alizarin red staining showed that BMSCs derived from HPP patients transfected with ALPL had increased capacity to form mineralized nodules when cultured under osteo-inductive conditions (a). Western blot analysis showed HPP patient derived BMSCs treated with Lentivirus-overexpressing ALPL had increased expression of osteogenic related proteins RUNX2 and Sp7 compared to HPP patient derived BMSCs (b). (c, d) HPP patient derived BMSCs treated with Lentivirus-overexpressing ALPL showed a decreased number of Oil red O-positive cells compared HPP patient derived BMSCs when cultured under adipo-inductive conditions (c) and upregulation of the adipogenic related proteins PPARγ2 and LPL, as assessed by Western blot (d). β-actin was used as a protein loading control. Representative results were from three independent experiments. *P<0.05, **P

< 0.01, ***P < 0.001

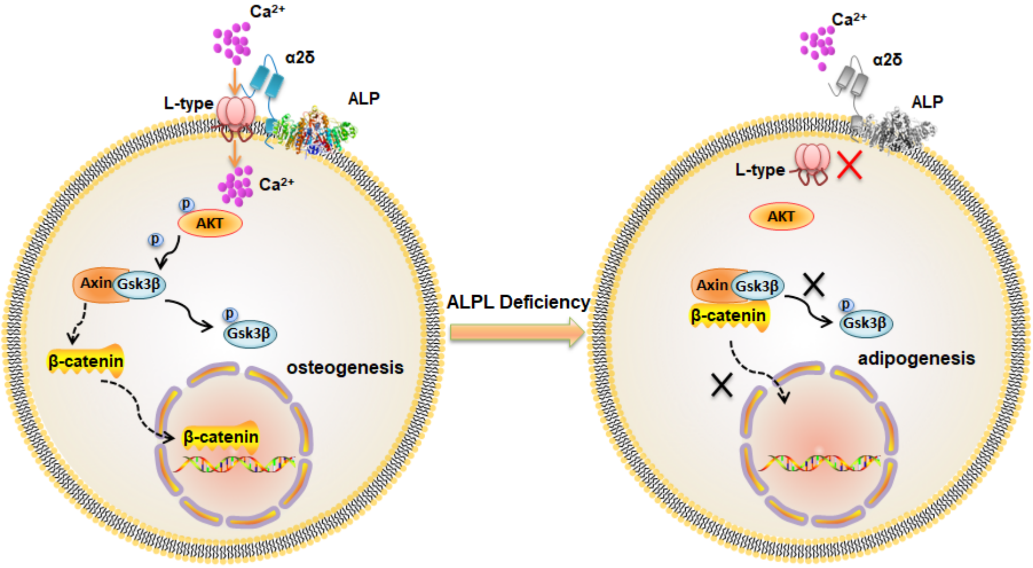

Figure S9. Graphic Summary shows the mechanisms of ALPL in regulating BMSC lineage commitment. ALPL maintains lineage differentiation of BMSCs through protein association with α2δ subunit of L-type Ca^{2+} channels and inhibiting the internalization of L-type Ca^{2+} channels, thus increases Ca^{2+} influx. Increased Ca^{2+} influx promotes phosphorylation of AKT and GSK, then activates Wnt/β-catenin pathway and regulates lineage commitment of BMSCs.

Table S1. Baseline table of two HPP patients

	A1	A2	Normal
**Sex**	Male	Female	/
**Age**	10	2.5	/
**Mutation site**	c.551G>A, p.R184Q	c.98C>T, p.A33V<br>c.422C>A, p.T141N	no
**History of Drug Allergy**	0	0	/
**Bone strength**	P=38	P=16	P≥50
**Serum alkaline Phosphatase activity**	80±1 IU/L	25±1 IU/L	244±17 IU/L
